# GC-derived exosomal circMAN1A2 promotes cancer progression and suppresses T-cell antitumour immunity by inhibiting FBXW11-mediated SFPQ degradation

**DOI:** 10.1186/s13046-025-03288-9

**Published:** 2025-01-25

**Authors:** Yikai Shen, Jie Lin, Tianlu Jiang, Xusheng Shen, Ying Li, Yiwang Fu, Penghui Xu, Lang Fang, Zetian Chen, Hongxin Huang, Yiwen Xia, Zekuan Xu, Linjun Wang

**Affiliations:** 1 Gastric Cancer Center, The First Affiliated Hospital of Nanjing Medical University, Nanjing, Jiangsu Province China; 2 Jiangsu Key Lab of Cancer Biomarkers, Prevention and Treatment, Collaborative Innovation Center for Cancer Personalized Medicine, Nanjing Medical University, Nanjing, Jiangsu Province China; 3 The Affiliated Wuxi People’s Hospital of Nanjing Medical University, Wuxi Medical Center, Nanjing Medical University, Wuxi People’s Hospital, Wuxi, Jiangsu Province China

**Keywords:** Gastric cancer, Exosomes, circMAN1A2, Antitumour immunity

## Abstract

**Background:**

Exosomes, as extracellular membrane vesicles, play important roles in intercellular communication and can influence tumour progression. Circular RNAs (circRNAs) have been reported in various malignancies and are also important components of exosomes. However, the role of exosomal circRNAs in gastric cancer (GC) progression has not been completely clarified.

**Methods:**

The exosomal circRNAs enriched in GC were identified using exosomal circRNA sequencing. The biological function of circMAN1A2 in GC was investigated using a series of in vitro and in vivo experiments. PKH-67 staining was used to label the exosomes. The molecular mechanism of exosomal circMAN1A2 was investigated via mass spectrometry, immunoprecipitation, Western blot, and single-cell RNA-sequencing data analyses.

**Results:**

In our study, we determined that circMAN1A2 (hsa_circ_0000118) was enriched in GC-derived exosomes. Higher circMAN1A2 expression was related to poor survival in GC patients (HR = 2.917, *p* = 0.0120). Exosomal circMAN1A2 promoted GC progression in vitro and in vivo and suppressed the antitumour activity of T cells. Moreover, circMAN1A2 bound to SFPQ in GC cells and T cells, promoting the G1/S phase transition of the cell cycle in GC cells while inhibiting the activation of the T cell receptor signalling pathway in T cells to decrease antitumour activity. Mechanistically, circMAN1A2 competed with FBXW11 for binding to SFPQ, preventing FBXW11-mediated k48-linked ubiquitination and SFPQ protein degradation, thereby stabilizing SFPQ expression.

**Conclusions:**

Our work confirms the critical role of exosomal circMAN1A2 in the progression and immunosuppression of GC. This novel axis of circMAN1A2-SFPQ provides new insights into exosomal circRNA-based GC diagnostic and therapeutic strategies.

**Supplementary Information:**

The online version contains supplementary material available at 10.1186/s13046-025-03288-9.

## Introduction

Gastric cancer (GC) is one of the most common tumours worldwide, with nearly 968,000 new cases and 660,000 deaths each year, ranking fifth among all malignancies worldwide in terms of incidence and mortality rates [1]. Although many new advances have been made in the diagnosis and treatment of GC, the overall prognosis of GC patients is poor, with a five-year overall survival rate of only approximately 30–35%. Therefore, there is an urgent need to investigate new molecular mechanisms and therapeutic strategies to improve the prognosis of GC patients.

Exosomes are a type of extracellular vesicle (EV) with a diameter of approximately 30–150 nm that can be secreted by a variety of cell types [2]. Exosomes are capable of carrying and transporting a range of molecules, including proteins, lipids, RNA and DNA, and play important roles in intercellular communication [3, 4]. Numerous studies have demonstrated that tumour-derived exosomes are involved in the formation and progression of a wide range of tumours, including remodelling of the tumour microenvironment (TME), regulation of antitumour immunity and drug resistance [4, 5]. Circular RNA (circRNA), a special type of RNA that is generated mainly by back-splicing of precursor mRNAs (pre-mRNAs), is also an important component of exosomes [6, 7]. CircRNAs are thought to be extremely stable, conserved and impervious to the majority of RNA decay mechanisms [8, 9]. Mounting evidence indicates that circRNAs can serve as potential biomarkers in many malignancies [10–12].

Recent studies on tumour immune-related mechanisms have concluded that immune and cancer cells frequently interact to affect the occurrence and progression of cancer [13]. In general, stronger antitumour immunity is associated with a better prognosis for patients [14]. A variety of tumour-infiltrating immune cells, such as macrophages, T cells and NK cells, are present in the GC tumour microenvironment [15].

Tumour-cell derived exosomes can carry immunosuppressive factors that inhibit the activity of immune cells and help tumours evade immune surveillance [16, 17]. In addition, exosomes can also regulate the immune microenvironment by binding to target cells and delivering signalling molecules to other immune cells [18].

Growing evidence indicates that circRNAs affect antitumour immunity by influencing immune cell function [19, 20]. However, the role of exosomal circRNAs in tumour immunity is not yet completely understood.

In this study, we investigated the role of differentially expressed exosomal circRNAs in immune regulation in the GC tumour microenvironment, aiming to provide ideas for GC immunotherapy.

## Methods

### Patients and clinical samples

Tissue samples used in this study, including GC and adjacent normal tissues, were obtained from patients who underwent radical GC resection in the Gastric Cancer Center at the First Affiliated Hospital of Nanjing Medical University between 2017 and 2018. Before surgery, no patients had adjuvant chemotherapy. Each participant provided written informed consent, and the study was authorized by the Nanjing Medical University Ethics Committee.

### Cell lines and culture

The HEK293T cells, human T cell line Jurkat, GC cell lines (validated by short tandem repeats for DNA fingerprinting) including HGC27, MKN45, KATOIII, SNU1, MKN28 and AGS were all purchased from Shanghai Institutes for Biological Sciences. HGC27, MKN45, SNU-1, MKN28 and Jurkat cells were cultured in RPMI-1640 medium (WISENT, Canada), HEK293T and KATOIII were cultured in Dulbecco’s modified Eagle medium (DMEM; WISENT, Canada) and AGS cells were cultured in F-12 K medium (WISENT, Canada). 10% fetal bovine serum (FBS; Gibco, USA) and 1% penicillin/streptomycin antibiotics (Gibco, USA) were added to the nutrient medium. All the cells were incubated in a humidified environmental incubator (5% CO2, 37℃).

### Exosome isolation and identification

Cells were cultured in aforementioned medium until they were 70% confluent. Then, the medium supplemented with 10% exosome-depleted FBS was employed for the further cell culture. After 48 h, the cell culture supernatant (approximately 15 mL) was collected. Subsequently, the supernatant was successively centrifuged at 300 × g, 4℃ for 10 min, 2,000 × g, 4℃ for 10 min, and 10,000 × g, 4℃ for 30 min to remove cells, dead cells and cell debris. The pellet was collected by centrifugation at 100,000 × g, 4℃ for 90 min. After resuspension in PBS, the aforementioned centrifugation process was repeated to obtain exosomes.

The quantity and concentration of exosomes were measured using the NanoSight LM10 instrument (Malvern, UK). Transmission electron microscopy (TEM; JEOL, Japan) was used to observe the shape of exosomes. The BCA protein assay kit (Thermo Fisher Scientific, USA) was used to measure the protein concentration of exosomes, then Western blot was employed to identify the expression of exosome signature markers (TSG101, CD81, Alix and CD9) and calnexin (the endoplasmic reticulum membrane protein).

### Exosome labeling and tracking

The green fluorescent marker PKH67 (Umibio, China) was employed to label the exosomes that were collected. Briefly, 50 μL of dye working solution was added into 50 μg of exosomes. After a 10-min dark incubation period, staining was finished. After repeating the exosome separation and purification procedures and removing the dye, differential centrifugation was used to collect fluorescently labeled exosomes.

Jurkat cells were seeded on a Nunc Glass Bottom Dish (Thermo Fisher Scientific, USA), incubated with complete medium overnight. The second day, PKH67-labeled GC-exosomes were added into the dish. After co-incubated for eight hours, cells were collected by centrifugation and the supernatant was discarded. Cells were resuspended in PBS, centrifuged to remove PBS, resuspended in 4% paraformaldehyde, and fixed at room temperature for 10 min. After centrifugation to remove paraformaldehyde, cells were washed using PBS for 3 times. Then the cells were resuspended with DAPI, and incubated for 5 min at room temperature in a dark chamber. Cells were washed using PBS again, then resuspended in PBS and dropped onto the slide. Finally, put on the coverslip and leave to dry, protected from light. A fluorescence microscope was used to observe and analyze the distribution of exosomes.

### RNA extraction and quantitative real-time polymerase chain reaction (qRT-PCR)

Total RNA was extracted from cells or tissues using TRIzol reagent (Invitrogen, USA). NE-PER Nuclear and Cytoplasmic Extraction Reagents (Thermo Fisher Scientific, USA) was used to separate the nuclear and cytoplasmic fractions. NanoDrop ND-2000 spectrophotometer (Thermo Fisher Scientific, USA) was used to assess the concentration and quality of the extracted RNA. CircRNA and mRNA were reverse-transcribed into cDNA using PrimeScript RT Master Mix Kit (TaKaRa, Japan). According to the manufacturer’s protocol, FastStart Universal SYBR Green Master Kit (Roche, Germany) and 7500 Real-Time PCR System (Applied Biosystems, USA) were used to carry out qRT-PCR. GAPDH was utilized as an internal control for circRNA and mRNA. For exosomal circRNA, Caenorhabditis elegans miR-39 (5ʹ-UCACCGGGUGUAAAUCAGCUUG-3ʹ, 5 fmol/μL) was added as an exogenous control. The 2^−ΔΔCT^ method was used to calculate the relative expression levels. The primers used are listed in Supplementary file 1.

### Protein extraction and western blot

RIPA lysis buffer was employed to extract total protein. Protein concentration was assessed with the BCA protein assay kit (Leagene Biotechnology, China). Protein lysates were separated by SDS-PAGE and then transferred onto polyvinylidene difluoride (PVDF) membranes. The membranes were blocked for 2 h at room temperature using 5% evaporated milk, and then they were incubated with primary antibodies at 4℃ for about 12 h. The membranes were then three times washed by TBST and incubated at room temperature for 2 h with secondary antibodies conjugated with HRP. Finally, after dripping ECL chemiluminescent reagents (Millipore, USA) onto the membranes, the Tanon-4600 Imaging System (Biotanon, China) was used to expose the blots and acquire the images. The antibodies used in this study are listed in Supplementary file 1.

### Statistical analysis

SPSS 25.0 (IBM, SPSS, IL, USA) and GraphPad Prism, version 9.5 (GraphPad Software, CA, USA) were employed to carry out statistical analyses. All experiments were repeated at least 3 times. Quantitative data were exhibited as mean ± standard deviation (SD). Student’s t-test was employed to measure statistical significance between two groups, while analysis of variance (ANOVA) was used to evaluate the differences between multiple groups. The correlation between circMAN1A2 expression and clinicopathological parameters was calculated using the Chi-square test. Kaplan–Meier curves and log-rank test were used for survival analysis. *p* < 0.05 was defined as statistically significant (*p < 0.05; **p < 0.01; ***p < 0.001).

Full material and methods were described in Supplementary file 2.

## Results

### Bioinformatics analysis of differentially expressed exosomal circRNAs in human GC tissues

**Fig. 1:**
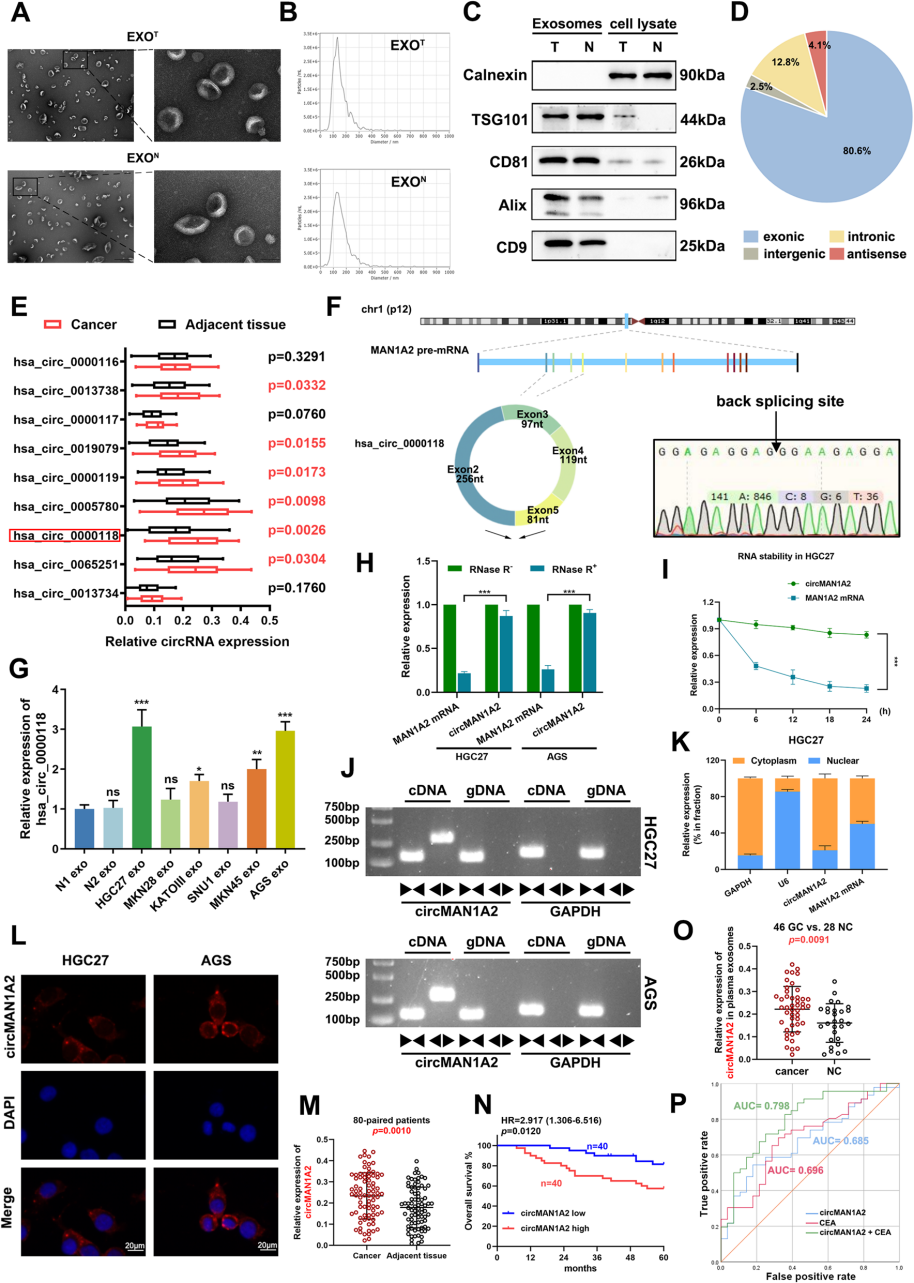
Bioinformatics analysis of differentially expressed exosomal circRNAs in human GC tissues. **A** Extracellular vesicles with double membranes, ranging in diameter from 30 to 150 nm, extracted from GC tissue and normal control were observed using electron microscopy. Scale bar: 500 nm (left), 100 nm (right). **B** Nanoparticle tracking analysis (NTA) revealed that the diameters of most purified exosomes were greater than 100 nm. **C** Extracted exosomes and cell lysate were subjected to Western blot analysis for signature exosomal markers (TSG101, CD81, Alix and CD9) and an endoplasmic reticulum membrane protein (calnexin), respectively. **D** The origin and distribution of all 33,997 circRNAs detected by exosomal circRNA sequencing. **E** Relative expression of the 9 circRNAs at the transcriptional level in 40 pairs of GC and adjacent normal tissues from GC patients. **F** circMAN1A2 (hsa_circ_0000118) was spliced from exons 2,3,4,5 of the precursor mRNA transcribed from the MAN1A2 gene located on chromosome 1, forming a circular transcript of 553 nt in length. Sanger sequencing confirmed the head-to-tail back-splicing of circMAN1A2. **G** The expression levels of circMAN1A2 in six different human GC cellular exosomes (HGC27 exo, MKN28 exo, KATOIII exo, SNU1 exo, MKN45 exo, and AGS exo). Data were normalized to the expression levels of circMAN1A2 in exosomes derived from normal gastric mucosal tissue. **H** qRT-PCR analysis of the level of circMAN1A2 and linear MAN1A2 mRNA after treatment with RNase R in HGC27 and AGS. **I** Relative levels of circMAN1A2 and MAN1A2 mRNA were measured by qRT-PCR in HGC27 treated with Actinomycin D for different periods of time. **J** The divergent primers detected circMAN1A2 in cDNA but not in gDNA, GAPDH was used as a negative control. **K** Relative levels of GAPDH (positive control for cytoplasmic fraction), U6 (positive control for nuclear fraction), circMAN1A2, and MAN1A2 mRNA from cytoplasmic and nuclear fractions in HGC27. **L** Fluorescence in situ hybridization (FISH) was conducted to confirm the sub-cellular localization of circMAN1A2 in HGC27 and AGS. Scale bar: 20 μm. **M** Relative circMAN1A2 expression at the transcriptional level in 80 paired GC and adjacent tissues. **N** Overall survival analysis based on the circMAN1A2 expression level in 80 GC patients. The median circMAN1A2 expression level was used as a cutoff. **O** The comparison of circMAN1A2 expression levels in plasma exosomes of GC patients and individuals without GC. **P** ROC curves of plasma exosomal circMAN1A2, serum CEA, and the combination of the two indicators (circMAN1A2: AUC = 0.685, 95% CI: 0.563–0.806, *p* = 0.008; CEA: AUC = 0.696, 95% CI: 0.575–0.818, *p* = 0.005; circMAN1A2 + CEA: AUC = 0.798, 95% CI: 0.693–0.903, *p* < 0.001). Graph represents mean ± SD; *p < 0.05, **p < 0.01, and ***p < 0.001

To analyse the differential expression of circRNAs in exosomes between gastric cancer and normal gastric mucosal epithelial tissues, we isolated exosomes from gastric cancer tissues and gastric mucosal epithelial tissues from healthy individuals via ultracentrifugation. As shown in Fig. 1a, typical extracellular vesicles with double membranes and diameters ranging from 30 to 150 nm were observed using electron microscopy. Nanoparticle tracking analysis (NTA) revealed that most of the diameters of the exosomes were greater than 100 nm (Fig. 1b). The detection of an endoplasmic reticulum membrane protein (calnexin) and signature exosomal markers (TSG101, CD81, Alix and CD9) verified the purity of the extracted exosomes (Fig. 1c).

Exosomal circRNA sequencing was subsequently performed. A total of 33,997 circRNAs were identified, and the origin and distribution of these circRNAs are shown in Fig. 1d. It can be seen that most of the circRNAs detected are formed from exons by back-splicing. The start**–**end length distributions of the genes corresponding to these circRNAs were queried using circAtlas (http://circatlas.biols.ac.cn) (Supplementary Fig. 1a). The chromosomal locus distributions of these circRNAs are shown in Supplementary Fig. 1b. Then, we focused on circRNAs enriched in GC-derived exosomes. After obtaining the intersection, we identified 75 circRNAs (Supplementary Fig. 1c). These 75 circRNAs were distributed on chromosomes 1, 3, 10 and 13 (Supplementary Fig. 1d). A total of 9 circRNAs were identified in more than five databases (circAtlas, Uniform, circBase, circRNADb, deepbase2, and circpedia2). Relative expression of these 9 circRNAs at the transcriptional level in 40 pairs of GC and adjacent normal tissues were quantified using qRT**-**PCR (Fig. 1e and Supplementary Fig. 1e, f). The results demonstrated that the difference in the expression of hsa_circ_0000118 between GC and adjacent normal tissues was the most significant (*p* = 0.0026). Then, we examined the expression levels of hsa_circ_0000118 in exosomes derived from 20 pairs of GC and adjacent normal tissues and found that its expression was elevated in GC-derived exosomes (*p* = 0.0074, Supplementary Fig. 1g). Therefore, we further explored the role of hsa_circ_0000118 in the biological progression of GC.

### Identification of circMAN1A2 and the clinical features of circMAN1A2

Hsa_circ_0000118 (termed “circMAN1A2” in this study) was spliced from exons 2, 3, 4 and 5 of the precursor mRNA transcribed from the MAN1A2 gene located on chromosome 1 (UCSC data in NCBI). CircMAN1A2 formed a circular transcript of 553 nt (Exon 2, 256 nt; Exon 3, 97 nt; Exon 4, 119 nt; Exon 5, 81 nt). We confirmed the head-to-tail back-splicing of circMAN1A2 using Sanger sequencing (Fig. 1f).

Next, circMAN1A2 expression levels were validated in six different human GC cell lines (HGC27, MKN45, KATOIII, SNU1, MKN28, and AGS) and two gastric mucosal epithelial tissues from healthy individuals. The expression levels of circMAN1A2 were elevated to varying degrees in GC cells and exosomes (Fig. 1g and Supplementary Fig. 2a). Notably, the most significant differences in circMAN1A2 expression levels were observed in both the cells and exosomes derived form the HGC27 and AGS cell lines. These two cell lines were selected for subsequent studies.

We performed several experiments to identify the circular characteristics of circMAN1A2. First, as shown in Fig. 1h, circMAN1A2 was resistant to RNase R exonuclease digestion compared with the linear form of MAN1A2. Next, we used actinomycin D, an inhibitor of transcription, to measure the changes in the half-life of circMAN1A2 and MAN1A2 mRNAs in both the HGC27 and AGS cell lines. The results suggested that circMAN1A2 is more stable than MAN1A2 mRNA (Fig. 1i and Supplementary Fig. 2b). Next, we designed divergent and convergent primers to identify circMAN1A2. By employing complementary DNA (cDNA) and genomic DNA (gDNA) extracted from HGC27 and AGS cells as templates, we found that circMAN1A2 was amplified exclusively from cDNA but not gDNA, indicating that the possibility of genomic rearrangements or trans-splicing was excluded (Fig. 1j). These experiments clearly confirmed the circular structure of circMAN1A2.

We subsequently explored the subcellular localization of circMAN1A2 in HGC27 and AGS cells. Following nucleocytoplasmic separation, we probed the relative circMAN1A2 expression levels in the cytoplasm and nucleus of the cells using qRT**-**PCR. The results revealed that circMAN1A2 was predominantly expressed in the cytoplasm of HGC27 and AGS cells (Fig. 1k and Supplementary Fig. 2c). The fluorescence in situ hybridization (FISH) assay further confirmed that circMAN1A2 is mainly localized in the cytoplasm (Fig. 1l).

We performed further qRT**-**PCR to determine the relative circMAN1A2 expression levels in paired cancer and adjacent tissues from 80 GC patients. The results confirmed greater circMAN1A2 expression levels in cancer tissues compared with adjacent tissues (Fig. 1m; *p* = 0.0010). Next, we integrated and analysed the clinical information of these patients. Patients were divided into high and low expression groups according to the median circMAN1A2 expression level. Statistical analysis revealed that circMAN1A2 expression levels correlated with lymph node metastasis and TNM stage (Table 1; Lymph node metastasis: *p* = 0.024, TNM stage: *p* = 0.006). Subsequently, using Kaplan**–**Meier survival analysis, we found that patients with high circMAN1A2 expression levels had a significantly worse prognosis (Fig. 1n; HR = 2.917, 95% CI = 1.306–6.516, *p* = 0.0120).

**Table 1:** Correlation between circMAN1A2 expression and the clinicopathologic parameters of 80 GC patients

Clinicopathologic parameter	Number	Number of patients		*p* value
		**circMAN1A2**^**low**^	**circMAN1A2**^**high**^	
**Age**				
< 60 years	18	6	12	0.108
≥ 60 years	62	34	28	
**Gender**				
Male	59	32	27	0.204
Female	21	8	13	
**Tumour size**				
< 3 cm	19	12	7	0.189
≥ 3 cm	61	28	33	
**Tumour site**				
Proximal	34	15	19	0.366
Non-proximal	46	25	21	
**Lymph node metastasis**				
N0	34	22	12	0.024^*^
N1-N3	46	18	28	
**TNM stage**				
I-II	30	21	9	0.006^**^
III	50	19	31	
**Differentiation**				
Well/Moderately	29	17	12	0.245
Poorly	51	23	28	
**Neural invasion**				
Negative	35	20	15	0.260
Positive	45	20	25	

^*^p < 0.05

^**^p < 0.01

To assess the diagnostic value of circMAN1A2, we collected blood from 46 GC patients and 28 individuals without GC (non-GC). qRT**-**PCR revealed that circMAN1A2 expression levels in the plasma exosomes of GC patients were significantly elevated compared with that of non-GC individuals (Fig. 1o; *p* = 0.0091). The receiver operating characteristic (ROC) curve results suggested that plasma exosomal circMAN1A2 in combination with CEA, a commonly used clinical serum tumour marker for GC, is instructive for the diagnosis of GC patients (Fig. 1p; circMAN1A2: AUC = 0.685, 95% CI: 0.563–0.806, *p* = 0.008; CEA: AUC = 0.696, 95% CI: 0.575–0.818, *p* = 0.005; circMAN1A2 + CEA: AUC = 0.798, 95% CI: 0.693–0.903, *p* < 0.001).

In summary, circMAN1A2 expression was confirmed to be upregulated in GC tissues and plasma exosomes. High circMAN1A2 expression is correlated with poor prognosis in GC patients. Thus, circMAN1A2 exhibits potential as a clinically valuable biomarker for GC.

### CircMAN1A2 promotes GC cell proliferation and migration in vitro

To study the biological role of circMAN1A2 in GC progression, we constructed three small interfering RNAs (siRNAs) that target the back-splicing region of circMAN1A2. Among them, si-circ-1 and si-circ-3 successfully inhibited circMAN1A2 expression in HGC27 and AGS cells without affecting the expression levels of the linear form of MAN1A2 (Supplementary Fig. 3a, b). Additionally, we transfected both GC cell lines with a circMAN1A2 overexpression plasmid. qRT**-**PCR was used to verify the transfection efficiency (Supplementary Fig. 3c).

**Fig. 2:**
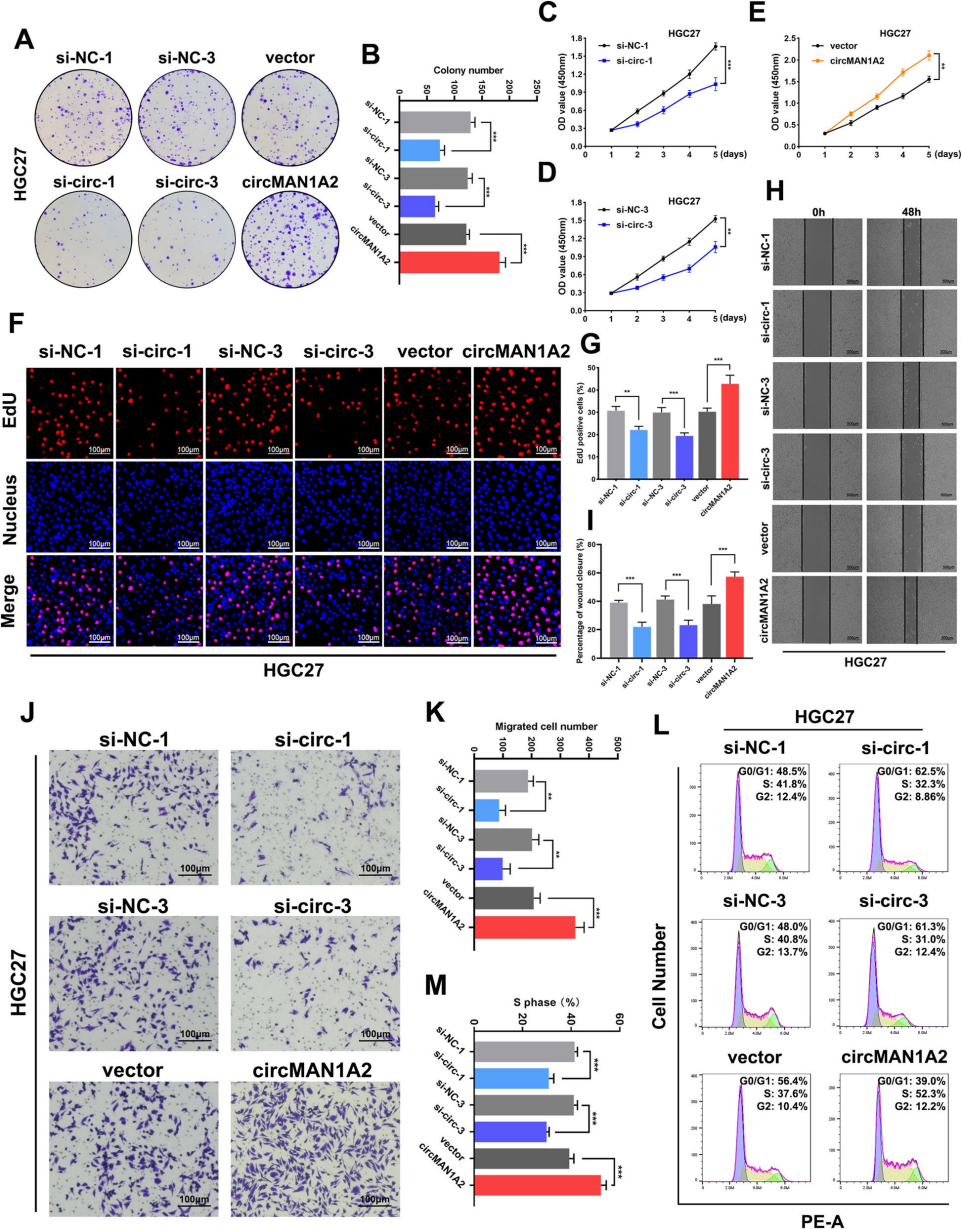
CircMAN1A2 promotes GC cell proliferation and migration in vitro. **A**, **B** The colony formation assay was performed to evaluate proliferation ability after upregulating or downregulating circMAN1A2 in HGC27 cells. **C-E** The CCK8 assay was performed to evaluate proliferation ability after upregulating or downregulating circMAN1A2 in HGC27 cells. **F**, **G** The EdU incorporation assay was performed to evaluate proliferation ability after upregulating or downregulating circMAN1A2 in HGC27 cells. Scale bar: 100 μm. **H**, **I** The wound healing experiment was performed to evaluate migration ability after upregulating or downregulating circMAN1A2 in HGC27 cells. Scale bar: 500 μm. **J**, **K** Transwell assay was performed to evaluate migration ability after upregulating or downregulating circMAN1A2 in HGC27 cells. Scale bar: 100 μm. **L**, **M** The effect of circMAN1A2 on modulating HGC27 cell cycle progression was evaluated by flow cytometry assay. Graph represents mean ± SD; *p < 0.05, **p < 0.01, and ***p < 0.001

We performed colony formation assays (Fig. 2a, b and Supplementary Fig. 4a, b), CCK-8 assays (Fig. 2c, d, e and Supplementary Fig. 4c, d, e) and EdU incorporation assays (Fig. 2f, g and Supplementary Fig. 4f, g) to explore the effects of circMAN1A2 expression on the proliferative capacity of GC cells. The results showed that the proliferation capacities of HGC27 and AGS cells transfected with siRNAs were significantly inhibited. In contrast, the proliferation capacity of HGC27 and AGS cells transfected with the circMAN1A2 overexpression plasmid was significantly increased. Moreover, wound healing experiments and Transwell assays were employed to explore the effects of circMAN1A2 on the migratory capacity of GC cells (Fig. 2h, i, j, k and Supplementary Fig. 4 h, i, j, k). The results indicated that circMAN1A2 promoted the migratory ability of GC cells; conversely, silencing circMAN1A2 expression significantly suppressed GC cell migratory capacity. We subsequently used flow cytometry to explore whether circMAN1A2 interferes with the cell cycle of HGC27 and AGS cells. The results revealed that interfering with circMAN1A2 expression decreased the percentage of S phase cells and increased the percentage of G0/G1 phase cells, and the opposite result was observed after circMAN1A2 overexpression (Fig. 2l, m and Supplementary Fig. 4l, m).

To further validate whether circMAN1A2 also exerts the biological function through entry into exosomes, we modulated the expression level of circMAN1A2 in HGC27 cells and then extracted cellular exosomes from different groups. We examined the extracted exosomes for circMAN1A2 expression levels (Supplementary Fig. 5a). Then, extracted exosomes were added to HGC27 cells for coculture. Colony formation, CCK-8, and EdU incorporation assays showed that the proliferative capacity of HGC27 cells was significantly reduced when coculturing with exosomes extracted form circMAN1A2 knockdown HGC27 cells. In contrast, exosomes derived from circMAN1A2-overexpressing HGC27 cells promoted the proliferation of HGC27 cells (Supplementary Fig. 5b, c, d, e, f). Furthermore, wound healing and Transwell assays demonstrated that coculturing HGC27 cells with exosomes from circMAN1A2-overexpressing HGC27 cells significantly enhanced cell migration, whereas co-culturing with exosomes from circMAN1A2-silenced HGC27 cells weakened cell migration (Supplementary Fig. 5 g, h, i, j). These findings suggest that exosomal circMAN1A2 promotes the malignant biological behaviors of GC cells.

Collectively, these findings confirmed that circMAN1A2 promotes GC cell proliferation and migration in vitro.

### CircMAN1A2 promotes GC proliferation and metastasis in vivo

We then performed animal experiments to determine whether circMAN1A2 could promote the malignant biological behaviours of GC in vivo.

**Fig. 3:**
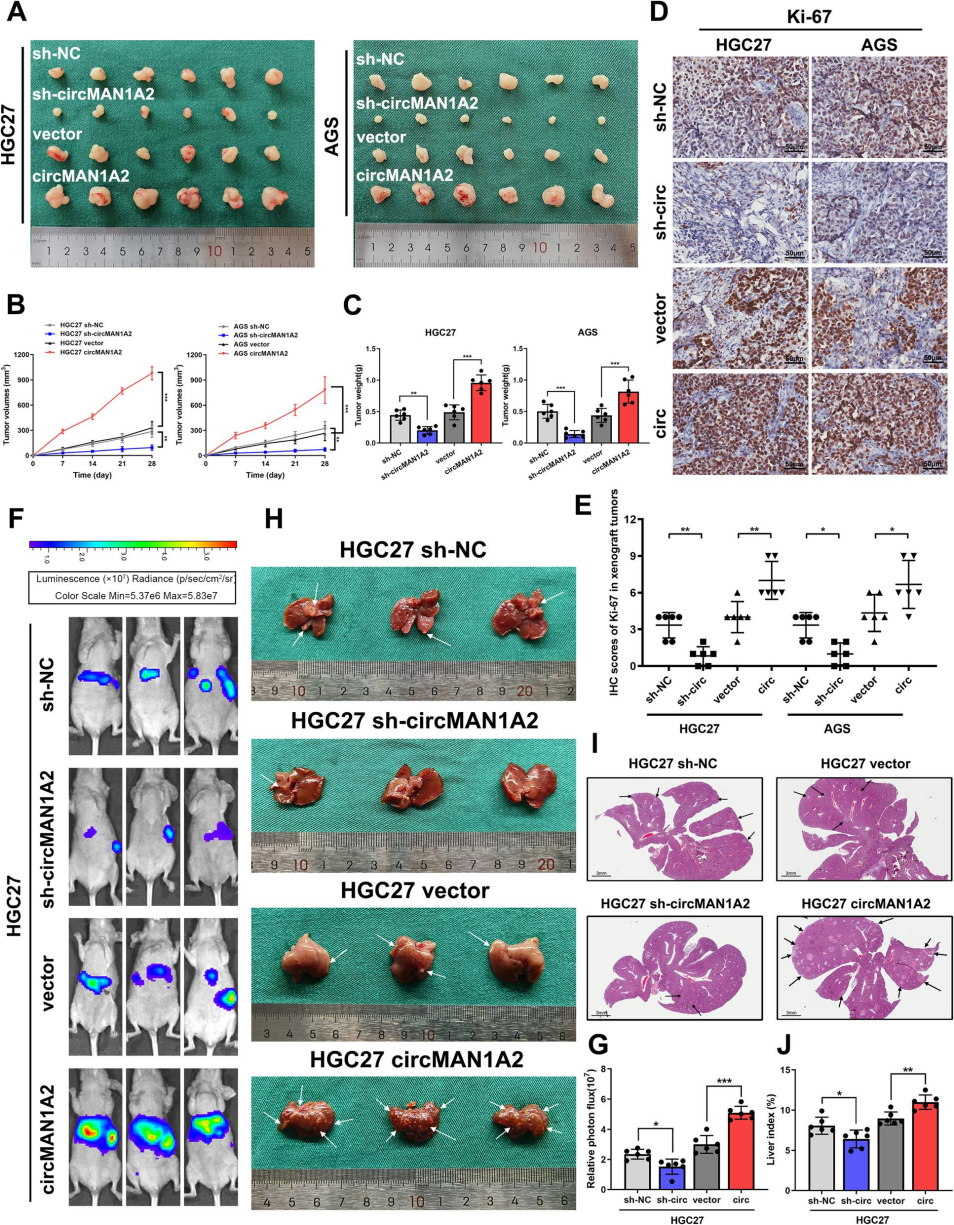
CircMAN1A2 promotes GC proliferation and metastasis in vivo. **A-C** The designated tumour cells were injected subcutaneously into nude mice, and xenograft tumours were collected 28 days later. Tumour volume was measured and calculated every week. Tumour weight was measured after 28 days. **D**, **E** Immunohistochemistry staining and IHC scores of Ki-67 in respective xenograft tumour tissues. Scale bar: 50 μm. **F**, **G** The representative Bioluminescence images of liver metastases after injecting tumour cells into the spleen of nude mice and quantification of these Bioluminescence images. **H** The representative photographs of liver tissues. White arrows point to liver metastases. **I** Representative images of HE staining of liver tissues. Black arrows point to liver metastases. Scale bar: 3 mm. **J** Liver index (liver weight/ body weight) of each group were calculated. Graph represents mean ± SD; *p < 0.05, **p < 0.01, and ***p < 0.001

HGC27 and AGS cells with stable circMAN1A2 knockdown by sh-circMAN1A2 were injected subcutaneously into nude mice, and the size of the subcutaneous tumours was observed weekly. Four weeks later, the nude mice were euthanized, and the subcutaneous tumours were removed. The size and weight of the tumours were measured for comparison of the differences between the groups. The results showed that the knockdown of circMAN1A2 limited the growth of subcutaneous xenograft tumours. In contrast, GC cells transfected with the plasmid overexpressing circMAN1A2 were able to promote subcutaneous tumour growth (Fig. 3a, b, c).

We then performed immunohistochemical staining. Ki-67 (a proliferation marker) staining was used to detect the proliferative capacity of the subcutaneous tumours (Fig. 3d). By calculation of IHC scores, we found that tumours with stable knockdown of circMAN1A2 showed weaker Ki-67 staining, while tumours in the cirMAN1A2-overexpression group had stronger Ki-67 staining. Thus we concluded that circMAN1A2 promotes GC proliferative capacity in vivo (Fig. 3e).

Next, we stably transfected the luciferase plasmids into the above cells to investigate the effect of circMAN1A2 on tumour metastasis. We constructed a liver metastasis (LM) model of GC in nude mice via the injection of GC cells into the spleen. Four weeks after surgery, we examined liver metastases in the nude mice via an IVIS Spectrum Imaging System (Fig. 3f, g). Then, we euthanized the nude mice, removed the liver tissues, and observed the number of liver metastases with the naked eye (Fig. 3h). Subsequently, the liver tissues were subjected to paraffin-embedded sectioning and H&E staining. By microscopic observation of H&E sections, we found that the number of GC-LM lesions was significantly suppressed after circMAN1A2 expression was knocked down. In contrast, when circMAN1A2 was overexpressed, the LM capacity increased (Fig. 3i, j). These results suggest that circMAN1A2 enhances GC metastasis. We further confirmed this finding using IHC staining of E-cadherin (the tumour aggressiveness marker) in subcutaneous tumours (Supplementary Fig. 6a, b).

Based on the above experiments, we confirmed that circMAN1A2 plays a biological role in promoting GC progression in vivo.

### CircMAN1A2 is packaged into exosomes via hnRNPA2B1 and delivered to T cells, which suppresses immune properties

We then investigated how circMAN1A2 is packaged into exosomes in GC cells and how it functions through exosomes.

First, we analysed the mass spectrometry (MS) data of circMAN1A2 pull-down experiments in HGC27 cells (Supplementary Fig. 7a). HnRNPA2B1, which has been reported to package a variety of RNAs into exosomes, attracted our attention (Supplementary Fig. 7b) [21, 22]. RIP**-**qPCR and RNA pull-down experiments were performed in HGC27 and AGS cells to validate the interaction between circMAN1A2 and hnRNPA2B1 (Supplementary Fig. 7c, d). Following the knockdown of hnRNPA2B1 expression in HGC27 and AGS cells, circMAN1A2 expression decreased significantly in the extracted exosomes (Supplementary Fig. 7e). These findings further confirmed that circMAN1A2 is encapsulated in exosomes through interactions with hnRNPA2B1.

**Fig. 4:**
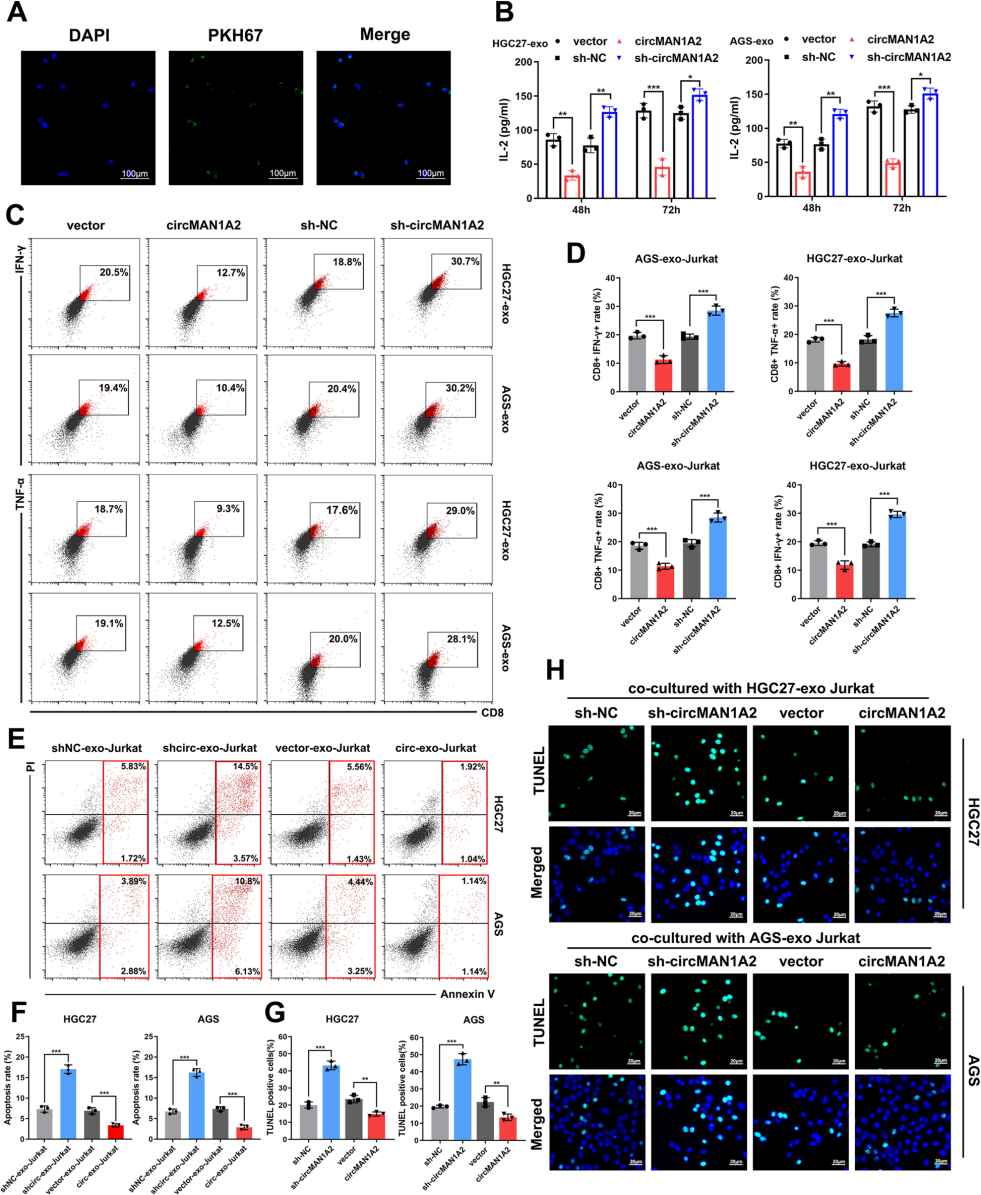
CircMAN1A2 is packaged into exosomes and delivered to T cells, suppressing the antitumour immunity. **A** HGC27-derived exosomes, labeled with PKH-67(green), were found to be taken up by Jurkat cells. Scale bar: 100 μm. **B** ELISA assays evaluated the secretion levels of IL-2 from Jurkat cells under the influence of GC-derived exosomal circMAN1A2. **C**, **D** Representative images of the flow cytometry assay showed the percentage of CD8 + IFN-γ + and CD8 + TNF-α + Jurkat cells under the influence of GC-derived exosomal circMAN1A2. **E**, **F** GC cells were cocultured with Jurkat cells for 24 h and then the apoptosis rates of GC cells were evaluated by flow cytometry, using annexin V-FITC and propidium iodide (PI) double labeling. **G**, **H** Representative images of TUNEL staining of GC cells after cocultured with different treatments of Jurkat cells. Scale bars: 20 μm. Graph represents mean ± SD; *p < 0.05, **p < 0.01, and ***p < 0.001

Exosomes secreted by tumour cells can influence immune infiltrating cells in the tumour microenvironment and thus modulate antitumour immunity [23, 24]. CD8^+^ T cells have long been considered among the most representative antitumour cells. Therefore, to investigate whether GC-derived exosomal circMAN1A2 affects CD8^+^ T cells, we performed further experiments. Exosomes extracted from HGC27 cells were stained with PKH67 and then cocultured with Jurkat cells. Exosomes can be taken up by Jurkat cells, suggesting that GC-derived exosomes may enter T cells to affect the tumour immune microenvironment (Fig. 4a). Therefore, we extracted exosomes from different groups of GC cells with circMAN1A2 overexpression or silencing and cocultured them with Jurkat cells. ELISAs verified that, compared with those derived from control cells, exosomes derived from circMAN1A2-overexpressing GC cells inhibited IL-2 secretion from Jurkat cells (Fig. 4b). Flow cytometry analysis revealed that the secretion of T-cell effector indicators, such as IFN-γ and TNF-α, was suppressed when Jurkat cells were cocultured with exosomes extracted from GC cells overexpressing circMAN1A2. In contrast, inhibition of circMAN1A2 expression in GC cells resulted in a significant increase in effector secretion by T cells (Fig. 4c, d). After coculturing the above treated Jurkat cells with GC cells for 24 h, we measured the degree of apoptosis in the GC cells using flow cytometry. Jurkat cells in the circMAN1A2-exo group inhibited the apoptosis of GC cells, whereas those in the sh-circMAN1A2-exo group exhibited the opposite trend (Fig. 4e, f). TUNEL staining further revealed that Jurkat cells, after being co-cultured with exosomes derived from circMAN1A2-silenced GC cells, showed a significant increase in TUNEL-positive GC cells when co-cultured with corresponding GC cells. In contrast, Jurkat cells co-cultured with exosomes from circMAN1A2-overexpressing GC cells exhibited a notable reduction in TUNEL-positive GC cells following co-culture with corresponding GC cells. These findings provided further evidence that circMAN1A2 inhibited the effector function of T cells (Fig. 4g, h).

From the above experiments, we confirmed that circMAN1A2 is encapsulated in exosomes through hnRNPA2B1 and that T cells can take up GC-derived exosomes. GC-derived exosomal circMAN1A2 affects the immune activation and effector function of T cells, thus regulating the antitumour immune response.

### CircMAN1A2 exerts its biological function by binding to SFPQ in GC cells and T cells

Based on the above findings, we preliminarily verified that circMAN1A2 promotes the malignant biological behaviour of GC and inhibits the antitumour immunity of T cells. Therefore, we explored specific molecular mechanisms involved.

**Fig. 5:**
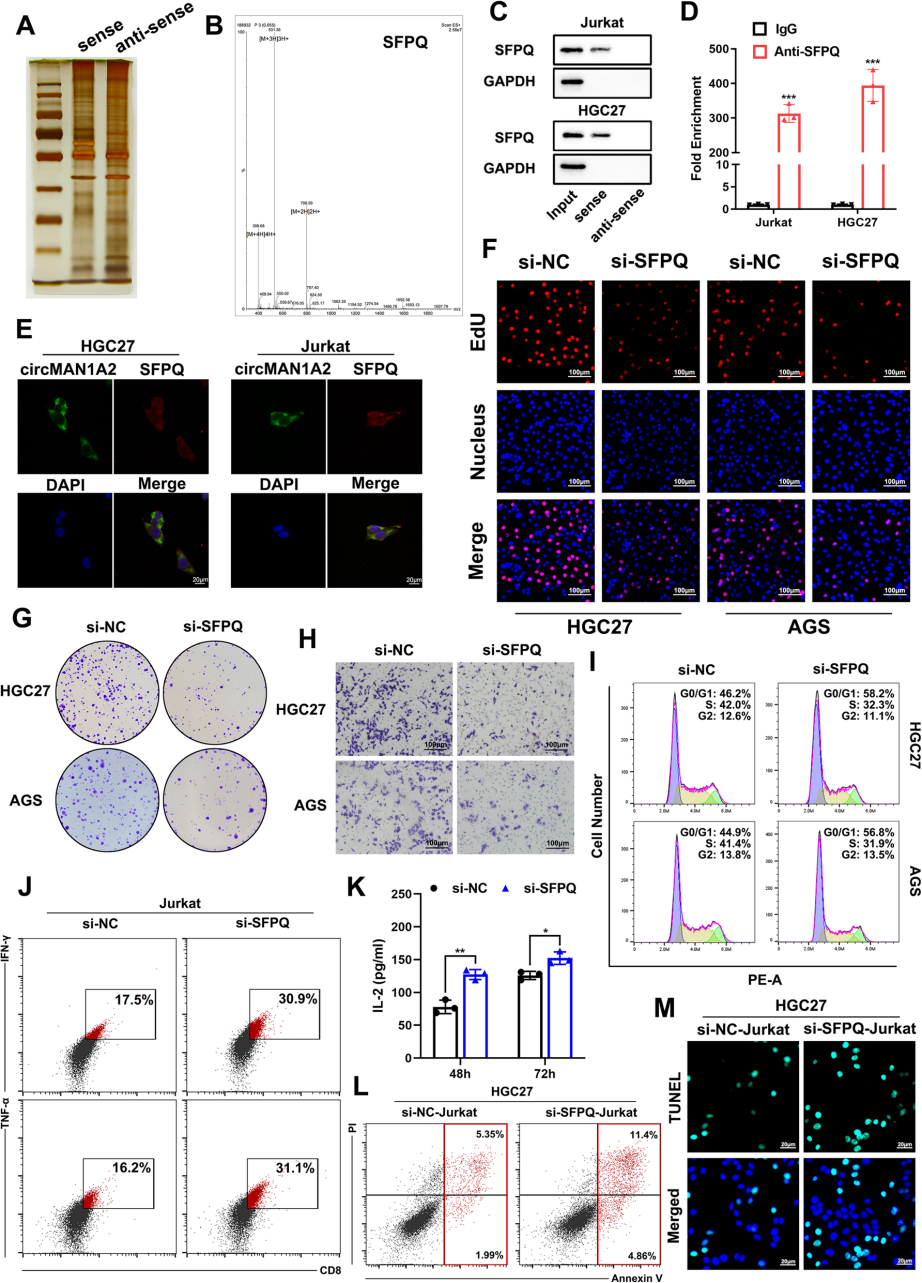
CircMAN1A2 exerts its biological function by binding to SFPQ in GC cells and T cells. **A** Protein bands detected by silver stain for mass spectrometry of the circMAN1A2-protein complex pulled down by sense or anti-sense circMAN1A2 in Jurkat cells. **B** The typical SFPQ peptide was identified in circMAN1A2-enriched proteins based on MS analysis. **C** RNA pull-down and Western blot assays were performed to confirm the interaction between SFPQ and circMAN1A2 in Jurkat and HGC27 cells. **D** RIP and qRT-PCR assays showed the interaction between SFPQ and circMAN1A2 in Jurkat and HGC27 cells, using IgG and SFPQ antibodies, *n* = 3. **E** Immunofluorescence-FISH was conducted to confirm the sub-cellular localization of circMAN1A2 and SFPQ in HGC27 and Jurkat cells. Scale bar: 20 μm. **F** The EdU incorporation assay was performed to evaluate proliferation ability after downregulating SFPQ in HGC27 and AGS cells. Scale bar: 100 μm. **G** The colony formation assay was performed to evaluate proliferation ability after downregulating SFPQ in HGC27 and AGS cells. **H** Transwell assay was performed to evaluate migration ability after downregulating SFPQ in HGC27 and AGS cells. Scale bar: 100 μm. **I** The effect of SFPQ on modulating GC cell cycle progression was evaluated by flow cytometry assay. **J** Representative images of the flow cytometry assay showed the percentage of CD8 + IFN-γ + and CD8 + TNF-α + Jurkat cells after downregulating SFPQ. **K** ELISA assays evaluated the secretion levels of IL-2 from Jurkat cells after downregulating SFPQ. **L** HGC27 cells were cocultured with Jurkat cells (downregulating SFPQ or not) for 24 h and then the apoptosis rates of HGC27 cells were evaluated by flow cytometry, using annexin V-FITC and propidium iodide (PI) double labeling. **M** Representative images of TUNEL staining of HGC27 cells after cocultured with different treatments of Jurkat cells. Scale bars: 20 μm. Graph represents mean ± SD; *p < 0.05, **p < 0.01, and ***p < 0.001

It has been reported that circRNAs function mainly by acting as competing endogenous RNAs (ceRNAs) and interacting with RNA-binding proteins (RBPs). We performed RNA pull-down experiments in HGC27 and Jurkat cells. AGO2 protein was not detected, suggesting that ceRNA may not be the main mechanism by which circMAN1A2 functions (Supplementary Fig. 8a). Next, we subjected the products of RNA pull-down in Jurkat cells to mass spectrometry (Fig. 5a). Combined with the mass spectrometry results from HGC27 cells (Supplementary Fig. 7a), SFPQ proteins were found to be present in both HGC27 and Jurkat cells (Fig. 5b).

SFPQ, splicing factor proline- and glutamine-rich, is a ubiquitous RNA-binding protein that is localized primarily in the nucleus and cytoplasm [25]. Dysregulation of SFPQ is often observed in neurological disorders such as Alzheimer's disease (AD) and amyotrophic lateral sclerosis (ALS) [26, 27]. However, reports suggest that SFPQ plays a significant role in tumourigenesis and progression by regulating the activation and repression of transcription, regulating splicing, and affecting the innate immune response [28, 29]. Therefore, we further explored its role in the biological progression of GC.

Using TIMER (Tumour Immune Estimation Resource, http://timer.cistrome.org/), we found that SFPQ was significantly negatively correlated with CD8 + T-cell infiltration (Supplementary Fig. 8b). RPIseq prediction revealed that SFPQ exhibited high binding potential with circMAN1A2 (Supplementary Fig. 8c) [30]. Then, we performed RNA pull-down experiments and RIP**-**qPCR experiments and found that circMAN1A2 interacts with SFPQ in both GC cells and Jurkat cells (Fig. 5c, d). Immunofluorescence-FISH confirmed the localization of circMAN1A2 and SFPQ in the cytoplasm of HGC27 cells and Jurkat cells (Fig. 5e). After interfering with SFPQ expression (Supplementary Fig. 9a), HGC27 and AGS cell proliferation and migration abilities were found to be inhibited by EdU incorporation assays, colony formation assays and Transwell assays (Fig. 5f, g, h and Supplementary Fig. 9b, c, d). HGC27 and AGS cell proliferation cycles were significantly arrested as detected by flow cytometry assays when silencing SFPQ expression (Fig. 5i and Supplementary Fig. 9e). In Jurkat cells, after downregulating SFPQ, the percentages of CD8 + IFN-γ + and CD8 + TNF-α + cells were increased (Fig. 5j and Supplementary Fig. 9f, g), and the secretion levels of IL-2 were elevated (Fig. 5k). When cocultured with SFPQ-silencing Jurkat cells, the apoptosis rates of HGC27 cells were found to be elevated compared with controls (Fig. 5l and Supplementary Fig. 9 h). TUNEL staining of HGC27 cells further showed the increase in TUNEL-positive cell numbers when cocultured with SFPQ-silencing Jurkat cells (Fig. 5m and Supplementary Fig. 9i), which indicated that the effector function was increased after interfering with SFPQ expression in Jurkat cells. Therefore, we suggested that SFPQ is a downstream molecule of circMAN1A2 in both HGC27 and Jurkat cells.

We subsequently aimed to investigate the structural regions of circMAN1A2 that interact with SFPQ. We utilized the catRAPID fragment module (http://s.tartaglialab.com/page/catrapid_group), an online site for RNA**–**protein binding prediction. In circMAN1A2, the 51–104, 126–179, 226–352 and 378–429 nt regions exhibit the potential to bind to SFPQ (Supplementary Fig. 10a). On the basis of these four regions, we designed four different circMAN1A2 truncation probes (Supplementary Fig. 10b). RNA pull-down assays were conducted with the full-length and truncated probes of circMAN1A2 to identify the specific areas that bind to SFPQ. We detected SFPQ proteins in the RNA pull-down assays of the experimental groups numbered 1, 3, 5, and 6 but not in the group numbered 4 (Supplementary Fig. 10c). Thus, we inferred that SFPQ mainly binds to the 226–352 nt region of circMAN1A2.

**Fig. 6:**
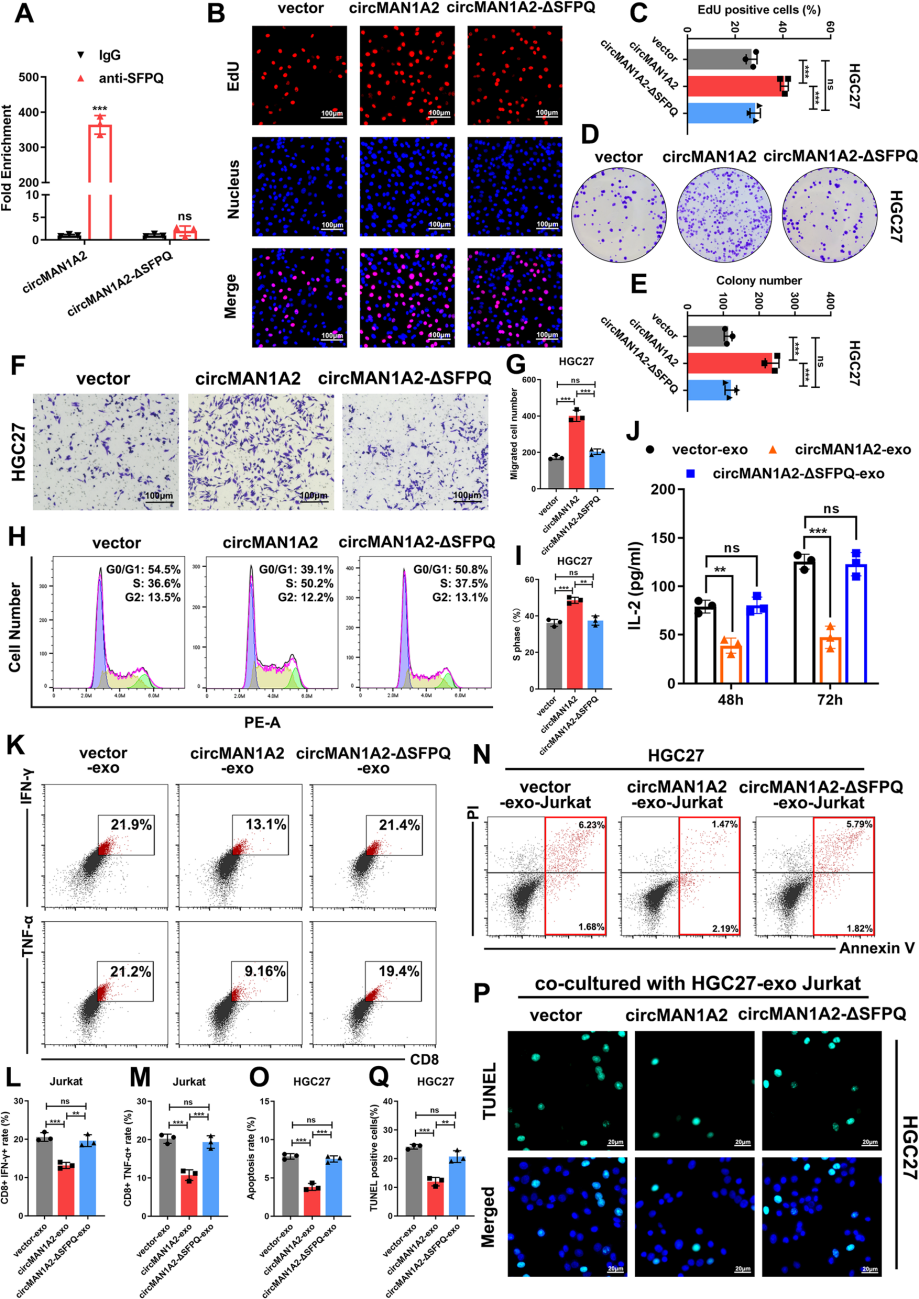
CircMAN1A2 interacts with SFPQ to promote GC progression and suppress immune activation and tumour killing effects of T cells. **A** RIP and qRT-PCR assays showed the interaction between SFPQ and circMAN1A2 but not circMAN1A2-△SFPQ in HEK293T cells, using IgG and SFPQ antibodies, *n* = 3. **B**, **C** The EdU incorporation assay was performed to evaluate proliferation ability after upregulating circMAN1A2 or circMAN1A2-△SFPQ in HGC27 cells. Scale bar: 100 μm. **D**, **E** The colony formation assay was performed to evaluate proliferation ability after upregulating circMAN1A2 or circMAN1A2-△SFPQ in HGC27 cells. **F**, **G** Transwell assay was performed to evaluate migration ability after upregulating circMAN1A2 or circMAN1A2-△SFPQ in HGC27 cells. Scale bar: 100 μm. **H**, **I** The effects of circMAN1A2 and circMAN1A2-△SFPQ on modulating HGC27 cell cycle progression were evaluated by flow cytometry assay. **J** ELISA assays evaluated the secretion levels of IL-2 from Jurkat cells after cocultured with exosomes extracted from circMAN1A2 or circMAN1A2-△SFPQ overexpressed HGC27 cells. **K-M** Representative images of the flow cytometry assay showed the percentage of CD8 + IFN-γ + and CD8 + TNF-α + Jurkat cells after cocultured with exosomes extracted from circMAN1A2 or circMAN1A2-△SFPQ overexpressed HGC27 cells. **N**, **O** HGC27 cells were cocultured with Jurkat cells (cocultured with exosomes extracted from circMAN1A2 or circMAN1A2-△SFPQ overexpressed HGC27 cells) for 24 h and then the apoptosis rates of HGC27 cells were evaluated by flow cytometry, using annexin V-FITC and propidium iodide (PI) double labeling. **P**, **Q** Representative images of TUNEL staining of HGC27 cells after cocultured with different treatments of Jurkat cells. Scale bars: 20 μm. Graph represents mean ± SD; *p < 0.05, **p < 0.01, and ***p < 0.001

We subsequently constructed circMAN1A2-ΔSFPQ and verified that it lost the ability to bind to SFPQ via RIP**-**qPCR experiments in HEK293T cells (Fig. 6a). A series of cellular experiments, such as colony formation assays, Transwell assays and cell cycle assays, revealed that circMAN1A2-ΔSFPQ does not affect the malignant biological progression of GC cells (Fig. 6b, c, d, e, f, g, h, i).

Next, we extracted exosomes from HGC27 cells overexpressing circMAN1A2 or overexpressing circMAN1A2-ΔSFPQ and cocultured them with Jurkat cells. The levels of secreted IL-2 in Jurkat cells were detected via ELISA, and the levels of secreted indicators of CD8 + T cells, such as IFN-γ and TNF-α, were detected using flow cytometry analysis. The results showed that circMAN1A2-ΔSFPQ does not have the ability to suppress CD8 + T-cell immune activation (Fig. 6j, k, l, m). In addition, the apoptosis levels of GC cells cocultured with the circMAN1A2-exo group Jurkat cells were reduced, whereas no significant differences were noted between the circMAN1A2-ΔSFPQ group and the control group (Fig. 6n, o). TUNEL staining also confirmed that circMAN1A2-ΔSFPQ reverses the effect of GC cell apoptosis inhibition by Jurkat cells in the circMAN1A2 group (Fig. 6p, q).

From the above experiments, we concluded that circMAN1A2 promotes GC progression by binding to SFPQ through its 226–352 nt region and that GC-derived exosomal circMAN1A2 inhibits immune activation and tumour killing effects by binding to SFPQ in T cells through its 226–352 nt region.

### Single-cell analysis confirmed that SFPQ promotes GC progression by regulating the cell cycle of GC cells and suppressing the antitumour immunity of T cells

**Fig. 7:**
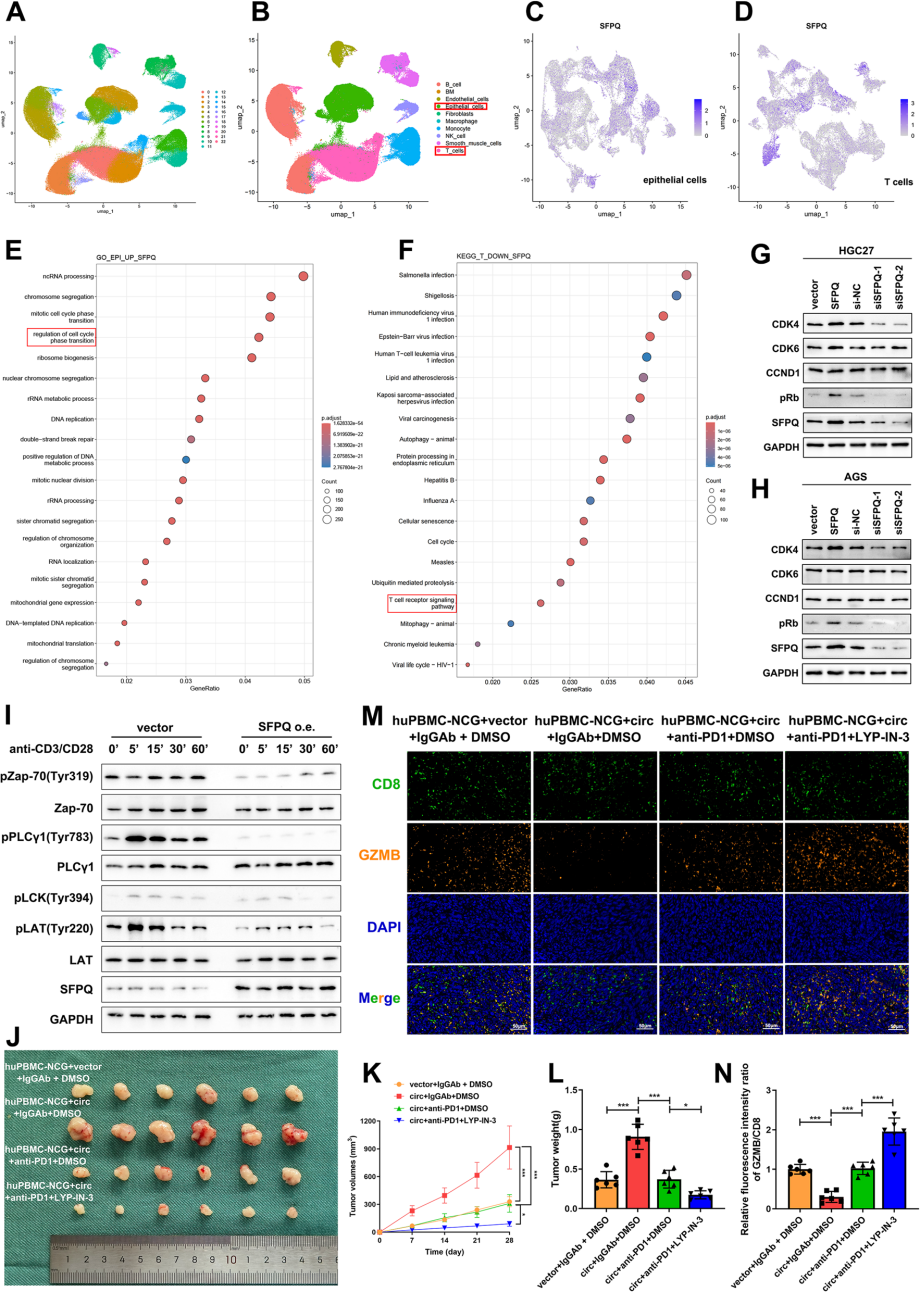
Single-cell analysis confirms that SFPQ promotes GC progression by regulating the G1/S phase transition of cell cycle and suppresses antitumour immunity of T cells by inhibiting the TCR signalling pathway. **A** Plotting of 23 cell clusters in single-cell RNA sequencing. **B** Plotting of 10 cell types after cell type annotation in single-cell RNA sequencing. **C** Distribution of SFPQ genes among epithelial cells. **D** Distribution of SFPQ genes among T cells. **E** Gene Set Enrichment Analysis of the differentially expressed genes in the SFPQ high expression group epithelial cell subpopulation. **F** Gene Set Enrichment Analysis of the differentially expressed genes in the SFPQ low expression group T cell subpopulation. **G**, **H** The expression levels of key proteins in G1/S phase progression (CCND1, CDK4, CDK6 and phosphorylated retinoblastoma) among GC cells with different treatments. **I** The phosphorylation levels of the key signalling proteins in the TCR signalling pathway among Jurkat cells with different treatments. **J**-**L** The designated GC cells were injected subcutaneously into huPBMC-NCG mice and different drugs were injected meanwhile. Xenograft tumours were collected 28 days later. Tumour volume was measured and calculated every week. Tumour weight was measured after 28 days. **M**, **N** Multiplex immunohistochemical (mIHC) detected the expression levels of CD8 and GZMB in tumour tissues of different groups. Scale bar: 50 μm. Graph represents mean ± SD; *p < 0.05, **p < 0.01, and ***p < 0.001

To investigate the mechanism of SFPQ function in GC and T cells, we employed single-cell analysis, which is able to assess the distribution of expression levels of each gene in different cell populations and can address cell-specific changes in the transcriptome. We integrated two large single-cell RNA sequencing (scRNAseq) datasets from the GEO database related to gastric cancer (GSE183904, GSE150290). A series of data quality control measures were first performed, including data integration, normalization and batch-effect correction. These procedures were followed by dimensionality reduction and clustering of the integrated data and cell identification on the basis of the expression of classical marker genes. The integrated data were clustered into 23 subpopulations and then classified into 10 cell types after cell type annotation (Fig. 7a, b). We selected the epithelial cell and T-cell subpopulations for further dimensionality reduction and clustering and visualized the distribution of SFPQ genes among them (Fig. 7c, d). We categorized epithelial cells and T cells into high and low SFPQ expression groups on the basis of their expression levels. We subsequently detected the differentially expressed genes (DEGs) and performed KEGG enrichment analyses. Among the enriched pathways, as shown in Fig. 7e, the DEGs in the high SFPQ expression group were enriched in “regulation of cell cycle phase transition” in the epithelial cell subpopulation. Moreover, the DEGs in the low SFPQ expression group were enriched in the “T-cell receptor (TCR) signalling pathway” in the T-cell subpopulation (Fig. 7f).

The cell cycle phase transition is tightly coordinated to ensure efficient cell cycle progression and genomic stability. Disruption of the cell cycle phase transition can lead to various diseases, including malignant tumours. Together, our findings confirmed that circMAN1A2 promotes the cell cycle progression of GC cells and that the GC cell cycle is arrested after circMAN1A2 expression is silenced (Fig. 2l and Supplementary Fig. 4 l). Similarly, cell cycle blockage in GC cells was observed when SFPQ was silenced (Fig. 5i). Interestingly, we found that changes in various phases of the cell cycle occur mainly in the G1 and S phases. Therefore, we hypothesized that SFPQ promoted the GC cell cycle by regulating the G1/S phase transition.

During the G1/S phase transition, Cyclin D1 (CCND1) is activated and binds Cyclin-dependent kinase 4/6 (CDK4/6), which phosphorylates the retinoblastoma protein (Rb). Then, the E2F transcription factor is released, contributing to the progression from G1 to S phase. Here, we examined the expression levels of key proteins involved in G1/S phase progression (CCND1, CDK4, CDK6 and phosphorylated retinoblastoma). We found that CDK4 and phosphorylated retinoblastoma (pRb) were upregulated after SFPQ overexpression. After SFPQ expression was silenced, CDK4 and pRb expression levels were significantly decreased (Fig. 7g, h). Thus, we hypothesized that SFPQ catalyses the G1/S phase transition and promotes GC cell proliferation.

The TCR signalling pathway plays indispensable roles in T-cell development, differentiation and the antitumour immune response. Recently, owing to the rise of cell therapies and immunotherapies, the TCR signalling pathway has attracted considerable attention in the field of cancer therapy. On the basis of the results of our single-cell analysis, we hypothesized that high SFPQ expression leads to blockade of the activation of the TCR signalling pathway, which subsequently affects the antitumour immunity of T cells. LCK, ZAP70, PLCγ1 and LAT phosphorylation are critical in the TCR signalling pathway [31–33]. We transfected a plasmid overexpressing SFPQ into Jurkat cells and stimulated the TCR using an anti-CD3/CD28 antibody. As shown in Fig. 7i, we found that the phosphorylation levels of these key signalling proteins in the SFPQ-overexpressing group were almost all reduced, whether at the basal level or under TCR stimulation at different time points. Therefore, we suggest that SFPQ affects the antitumour activity of T cells by inhibiting TCR signalling pathway activation.

Next, we verified that circMAN1A2 promotes GC progression in vivo by inhibiting TCR signalling pathway activation. We transplanted human peripheral blood mononuclear cells (PBMCs) into the NCG of severely immunodeficient mice and constructed a huPBMC-NCG mouse model for subsequent validation in animal experiments. Using subcutaneous tumour loading experiments, we found that, compared with those in the control group, the sizes of the xenograft tumours in the circMAN1A2-overexpressing group were significantly greater. However, anti-PD1 treatment reversed this trend, and the growth of subcutaneous tumours was further inhibited by the addition of the TCR receptor agonist LYP-IN-3 (Fig. 7j, k, l). Multiplex immunohistochemistry (mIHC) revealed that LYP-IN-3 increased the amount of GZMB secreted by cytotoxic T cells (Fig. 7m, n). The above experiments further suggest that circMAN1A2 affects the biological progression of GC by inhibiting the TCR signalling pathway.

Overall, we conclude that circMAN1A2 exerts its biological function by binding to SFPQ. Specifically, circMAN1A2 regulates the malignant biological behaviour of GC by contributing to the G1/S phase transition of the cell cycle. And in T cells, it regulates antitumour immune activity by inhibiting TCR signalling pathway activation.

### CircMAN1A2 interacts with the RRM1 domain of SFPQ and inhibits ubiquitin–proteasome-mediated degradation of SFPQ

We then explored the specific mechanisms by which circMAN1A2 interacts with SFPQ.

**Fig. 8:**
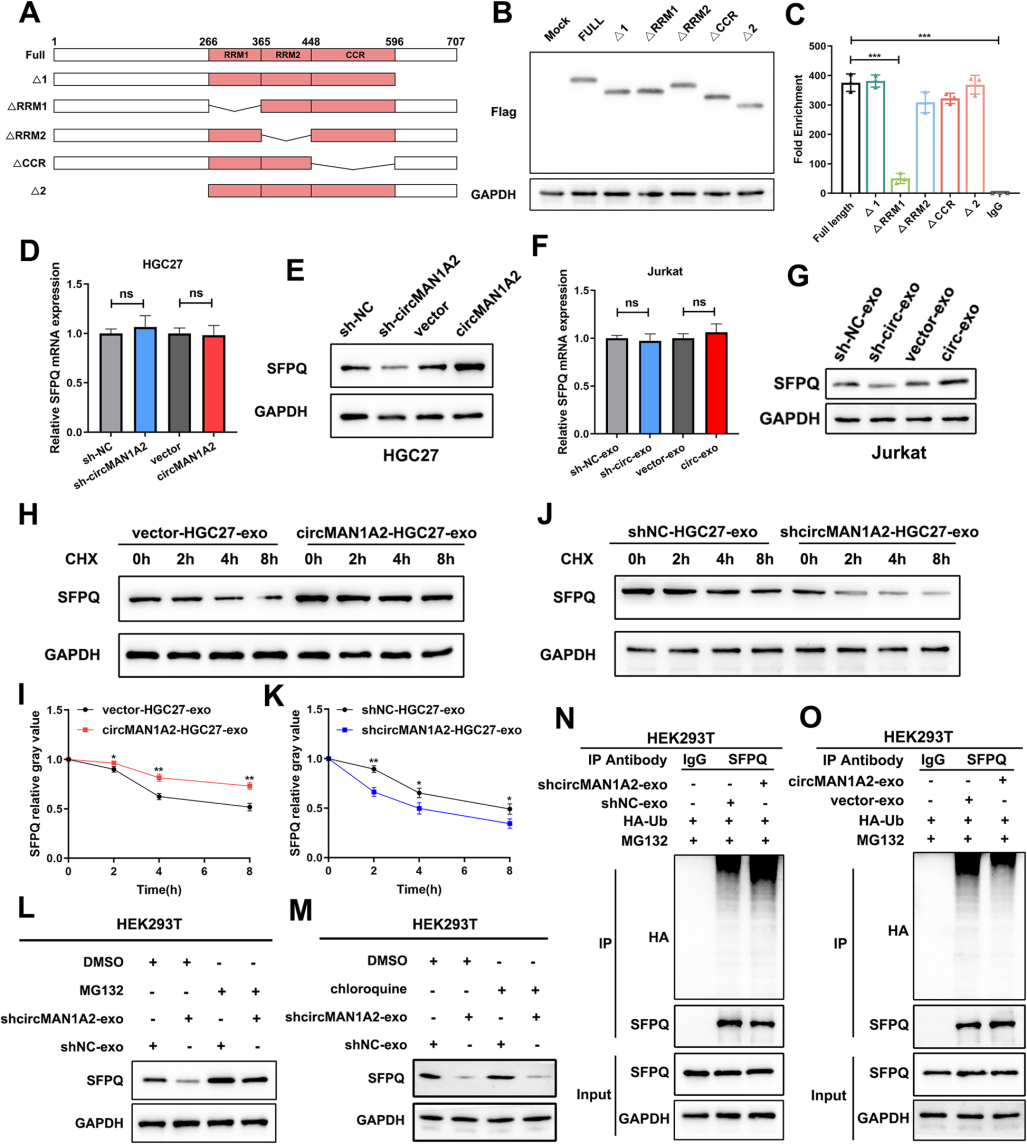
CircMAN1A2 interacts with the RRM1 domain of SFPQ and inhibits ubiquitin**–**proteasome**-**mediated degradation of SFPQ. **A** Truncated mutants of Flag-SFPQ designed according to the structural domains of SFPQ. **B** Verification of truncated mutants of Flag-SFPQ by Western blot. **C** RIP and qRT-PCR assays showed the interaction between different truncated mutants of SFPQ and circMAN1A2. **D** Relative SFPQ mRNA expression levels detected by qRT-PCR in different groups of HGC27 cells. **E** The SFPQ protein expression levels detected by Western blot in different groups of HGC27 cells. **F** Relative SFPQ mRNA expression levels detected by qRT-PCR in different groups of Jurkat cells. **G** The SFPQ protein expression levels detected by Western blot in different groups of Jurkat cells. **H–K** Western blot analysis of lysates from HEK293T cells, which cocultured with differently treated HGC27 cell-derived exosomes, and treated for various times with cycloheximide (CHX). **L**, **M** After cocultured with exosomes extracted from circMAN1A2-silenced HGC27 cells, HEK293T cells were treated with MG132 (10 µM) or chloroquine (100 μM) for 6 h. Then SFPQ expression levels were evaluated by Western blot. **N**, **O** HEK293T cells were cocultured with exosomes extracted from circMAN1A2 silenced or over-expressed HGC27 cells. IP assays were used to detect the poly-ubiquitination levels of SFPQ in HEK293T cells. Graph represents mean ± SD; *p < 0.05, **p < 0.01, and ***p < 0.001

First, we identified the specific domains of SFPQ necessary for the interaction with circMAN1A2. According to the structural domains of SFPQ available on the SMART website (https://smart.embl.de/), we constructed a series of plasmids stably expressing truncated mutants of Flag-SFPQ to transfect HEK293T cells (Fig. 8a). Western blot verificated these truncated muntants of Flag-SFPQ (Fig. 8b). RIP and qRT**-**PCR analysis suggested that full-length SFPQ, together with Δ1, ΔRRM2, ΔCCR, and Δ2, bound to circMAN1A2; however, ΔRRM1 was not able to interact with circMAN1A2 (Fig. 8c). These findings suggested that the RRM1 domain of SFPQ plays a crucial role in the circMAN1A2-SFPQ interaction.

Next, we found that SFPQ expression levels were significantly decreased in HGC27 cells with silenced circMAN1A2 expression. However, when circMAN1A2 was overexpressed, SFPQ expression levels clearly increased. Similarly, SFPQ protein levels in Jurkat cells increased after coculture with exosomes derived from HGC27 cells overexpressing circMAN1A2. In contrast, after coculture with exosomes derived from sh-cricMAN1A2 HGC27 cells, SFPQ expression levels in Jurkat cells appeared to decrease compared with that in the control group. However, SFPQ mRNA levels did not change significantly (Fig. 8d, e, f, g). On this basis, we confirmed that circMAN1A2 regulates SFPQ expression at the protein level rather than at the transcriptional level.

As described previously, we regulated circMAN1A2 expression levels in HGC27 cells and subsequently coincubated the extracted gastric cancer cell exosomes with HEK293T cells. The CHX chase assay revealed that, compared with the control group, the decline in SFPQ protein expression levels in HEK293T cells slowed after coculture with exosomes derived from HGC27 cells overexpressing circMAN1A2, leading to an extended half-life of SFPQ protein (Fig. 8h, i). The sh-circMAN1A2-exo group presented the opposite results (Fig. 8j, k). Moreover, the inhibition of SFPQ protein expression by sh-circMAN1A2-exo was reversed by the proteasome inhibitor MG132 but not by the lysosome inhibitor chloroquine (Fig. 8l, m). These results suggest that circMAN1A2 regulates SFPQ protein expression through proteasomal degradation.

Co-IP experiments revealed that, after coculture with exosomes extracted from circMAN1A2-silenced HGC27 cells, the poly-ubiquitination levels of SFPQ in HEK293T cells were significantly elevated compared with those in control cells. The circMAN1A2-overexpressing group presented the opposite results (Fig. 8n, o).

We confirmed that circMAN1A2 stabilizes SFPQ protein expression by inhibiting its ubiquitin**–**proteasome-mediated degradation.

### CircMAN1A2 competes with FBXW11 for binding to SFPQ, preventing FBXW11-mediated K48-linked SFPQ ubiquitination and protein degradation

**Fig. 9:**
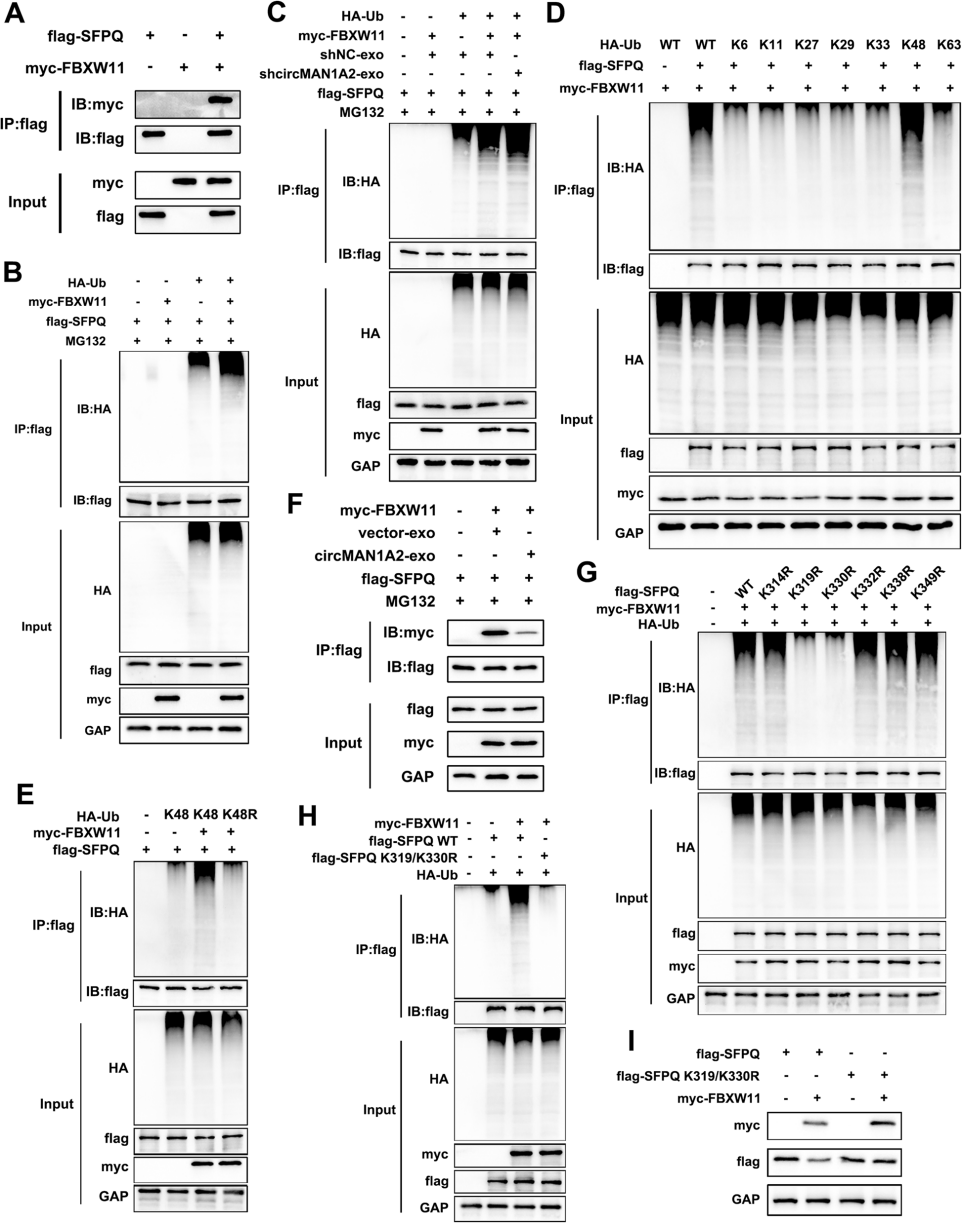
CircMAN1A2 competes with FBXW11 for binding to SFPQ, preventing FBXW11-mediated K48-linked SFPQ ubiquitination and protein degradation. **A** Co-IP experiments in HEK293T cells to validate the binding between SFPQ and FBXW11. **B** Co-IP assays showed that FBXW11 increased SFPQ ubiquitination levels. **C** Co-IP assays confirmed that SFPQ ubiquitination level increased in the HEK293T cells overexpressing FBXW11. And the ubiquitination level of SFPQ was further increased in FBXW11-overexpressed HEK293T cells, when cocultured with sh-circMAN1A2-HGC27 cell-derived exosomes. **D** HEK293T cells were transfected with SFPQ, FBXW11 and HA-tagged WT or ubiquitin mutants retaining the only specific K site (other six lysine residues were mutated to arginine), and then SFPQ ubiquitination levels were examined. **E** HEK293T cells were transfected with SFPQ, FBXW11 and HA-Ub (K48 or K48R) and subjected to ubiquitin assay. **F** Co-IP assays confirmed that circMAN1A2 inhibited the binding between FBXW11 and SFPQ. **G** HA-Ub, myc-FBXW11, along with flag-SFPQ (WT and its point mutants) were co-transfected to HEK293T cells, and then SFPQ ubiquitination levels were examined. **H** HA-Ub, myc-FBXW11, along with flag-SFPQ (WT or K319/330R mutant) were co-transfected to HEK293T cells, and then SFPQ ubiquitination levels were examined. **I** Western blot assays of HEK293T cells transfected with myc-FBXW11 and flag-SFPQ (WT or K319/330R mutant)

To further investigate which E3 ubiquitin ligases mediate SFPQ ubiquitination regulated by circMAN1A2, we performed a co-IP experiment in HEK293T cells using an SFPQ antibody, and the resulting protein samples were subject to silver staining (Supplementary Fig. 11a). MS analysis identified F-box and WD repeat domain containing 11 (FBXW11), which is an important component of the SCF (SKP1-CUL1-F-box) E3 ubiquitin ligase complex and is involved in the progression of several tumours (Supplementary Fig. 11b) [34, 35]. Further co-IP experiments confirmed the binding between SFPQ and FBXW11 (Fig. 9a). In addition, SFPQ ubiquitination levels were increased after FBXW11 overexpression, indicating that FBXW11 is an important E3 ubiquitin ligase for SFPQ (Fig. 9b).

Further experiments verified that FBXW11 overexpression significantly increased the level of SFPQ ubiquitination in HEK293T cells cocultured with sh-NC-HGC27 cell-derived exosomes. In comparison, the ubiquitination level of SFPQ was further increased in FBXW11-overexpressed HEK293T cells, when cocultured with sh-circMAN1A2-HGC27 cell-derived exosomes (Fig. 9c). Therefore, we conclude that FBXW11 acts as an E3 ubiquitin ligase for SFPQ, whereas circMAN1A2 inhibits the interaction between FBXW11 and SFPQ.

To clarify the type of FBXW11-mediated ubiquitination of SFPQ, we transfected various mutant forms of ubiquitin into HEK293T cells. As shown in Fig. 9d, FBXW11-mediated ubiquitination of SFPQ was detected only with ubiquitin mutants retaining the K48 site. As expected, SFPQ ubiquitination was prevented in the presence of K48R-Ub (Fig. 9e). These findings indicate that FBXW11 acts as an E3 ubiquitin ligase to mediate the K48-linked ubiquitination of SFPQ.

Next, we explored how circMAN1A2 regulates FBXW11-mediated ubiquitination of SFPQ. We cocultured HEK293T cells with exosomes derived from circMAN1A2-overexpressing HGC27 cells and then detected decreased binding of SFPQ to FBXW11 (Fig. 9f). In contrast, when HEK293T cells were cocultured with sh-circMAN1A2-HGC27-derived exosomes, the binding of SFPQ to FBXW11 was increased (Supplementary Fig. 11c). These findings indicate that circMAN1A2 competes with FBXW11 for binding to SFPQ.

To determine the potential lysine residue involved in SFPQ ubiquitination modulated by circMAN1A2, we used the Phosphosite website (https://www.phosphosite.org/) to predict SFPQ lysine ubiquitination sites within the binding domain of SFPQ and circMAN1A2. Among them, K314, K319, K330, K332, K338 and K349 were predicted to be ubiquitination sites with high confidence in the RRM1 domain of SFPQ. We found that SFPQ ubiquitination levels were significantly reduced by the K319R and K330R mutations (Fig. 9g). Further mutations of both the K319 and K330 sites were constructed (SFPQ-K319/K330R). SFPQ ubiquitination was almost completely eliminated when both the K319 and K330 sites were mutated (Fig. 9h, i). Thus, we concluded that the K319 and K330 sites were the major modification sites for FBXW11-mediated ubiquitination of SFPQ.

Therefore, these results suggest that circMAN1A2 competes with FBXW11 for binding to SFPQ and prevents FBXW11-mediated K48-linked SFPQ ubiquitination and protein degradation in GC and T cells, thus stabilizing SFPQ expression.

## Discussion

Although the systemic treatment of GC has improved recently, its cancer-related mortality rate remains high. Therefore, research into novel molecular mechanisms related to GC is urgently needed to provide new insights into the clinical treatment of GC. Exosomes are vesicles with a diameter of approximately 30–150 nm that are secreted from cells into extracellular regions and are present in a variety of biological fluids, such as blood, urine, and saliva. Exosomes are loaded with various cellular components (such as proteins, lipids, nucleic acids, and sugars) and play important roles in cell-to-cell communication, thus participating in many pathophysiological processes, including tumourigenesis and cardiovascular diseases. Many studies have reported that tumour-derived exosomes play important roles in tumour growth, immune regulation, and other processes. CircRNAs are formed by back-splicing of precursor mRNAs and are highly abundant and stable in exosomes. In recent years, the advancement in biotechnology and molecular medicine has led to growing interests in artificially constructed circRNA vaccines. Liu et al. constructed the circRNA vaccine which could prevent Zika virus infection without exacerbating dengue severity in mice [36]. Yue et al. developed a circRNA vaccine delivering multiple neuraminidase antigens that provides broad-spectrum protection against influenza in mice [37]. Wang et al. demonstrated that circRNAs expressing hepatocellular carcinoma-specific tumour neoantigens could promote dendritic cell (DC) activation in vitro and enhance DC-induced T-cell activation, thereby augmenting T-cell-mediated cytotoxicity against tumour cells. This circRNA vaccine exhibited remarkable tumour treatment and prevention in mouse models [38].

Exosomal circRNAs are thought to be potential markers for early cancer diagnosis and prognosis because their aberrant expression in tumour tissues is related to the development of several malignant tumours. For example, Wen et al. identified specific circRNAs (hsa-circ-0000367, hsa-circ-0021647, and hsa-circ-0000288) in the bile acid and serum exosomes of cholangiocarcinoma patients and constructed a model for the diagnosis and monitoring of early recurrence [39]. The tumour immune process involves the participation of multiple immune cells and reactive molecules. Exosomal circRNAs have been shown to play a significant role in immune modulation in a number of malignant tumours, including hepatocellular, ovarian, and colorectal cancer [40–42]. However, studies on the effects of exosomal circRNAs on tumour progression and antitumour immunity in patients with GC are relatively rare.

In this study, we employed exosomal circRNA sequencing to identify differentially expressed circRNAs in exosomes derived from GC compared with normal gastric mucosal epithelial tissues. A total of 33,997 circRNAs were identified, with the majority formed by exons through back-splicing. We focused on circRNAs that were enriched in GC-derived exosomes. By intersecting the data from three groups, we found that the expression of 75 circRNAs was significantly upregulated in GC-derived exosomes. Among them, 9 circRNAs were recognized in five or more databases (circAtlas, Uniform, circBase, circRNADb, deepbase2, and circpedia2). Quantitative analysis of their relative expression at the transcriptional level in GC and adjacent normal tissues, as well as in the corresponding exosomes, revealed that hsa_circ_0000118 (circMAN1A2) had the most significant difference. Therefore, we focused on circMAN1A2 in our study. circMAN1A2 is formed by the splicing of exons 2, 3, 4, and 5 of the precursor mRNA transcribed from the MAN1A2 gene located on chromosome 1. The stable head-to-tail loop structure of circMAN1A2 was experimentally verified. With further expansion of the sample size, the expression levels of circMAN1A2 were confirmed to be upregulated in GC cells and exosomes, compared to the control group. FISH and qRT-PCR confirmed the cytoplasmic localization of circMAN1A2. Survival analysis revealed that patients with high circMAN1A2 expression in tumour tissues had a poorer prognosis. Additionally, comparison of plasma exosomal circMAN1A2 levels between GC patients and control individuals showed significant upregulation in GC patients, suggesting its potential as a diagnostic marker for GC.

Next, we focused on the role of circMAN1A2 in GC progression and the potential mechanism involved. Multivariate analysis revealed that the circMAN1A2 expression level was correlated with the extent of lymph node metastasis and the TNM stage of GC patients. K**-**M survival analysis revealed that a high circMAN1A2 expression level was associated with poor prognosis. We discovered that plasma exosomal circMAN1A2 levels were considerably higher in GC patients than in normal individuals. ROC curve analysis indicated that plasma exosomal circMAN1A2 was useful in the diagnosis of GC (AUC = 0.685, 95% CI: 0.563–0.806, *p* = 0.008). Through experiments, we found that circMAN1A2 promoted the proliferation and migration of GC cells. Furthermore, we found that circMAN1A2 can be packaged into exosomes with the help of hnRNPA2B1.

Studies have reported that exosomes can affect immune cells in the tumour immune microenvironment. For example, exosomes released by gliomas have the capacity to influence the M2 polarization of macrophages in the tumour immune microenvironment to promote cancer progression [43]. The effector function of lymphocytes treated with tumour exosomes is impaired accordingly [44]. Tumour exosomes can also directly inhibit the killing function of NK cells in a T-cell-independent manner, thereby promoting immune escape [45]. Therefore, we investigated whether immune cells in the immune microenvironment are influenced by tumour-derived exosomal circMAN1A2, thereby affecting antitumour immunity in GC.

CD8 + T cells, also known as cytotoxic T lymphocytes (CTLs), play important roles in cellular immunity. Once CD8 + T cells are activated, they undergo a series of replication and differentiation processes that trigger a directed immunological response. Typically, CD8 + T cells induce cell apoptosis by releasing perforin and granzyme. Moreover, CD8 + T cells also kill target cells indirectly by releasing cytokines such as TNF-α and IFN-γ. In this study, we found that circMAN1A2 is transported by GC-derived exosomes and then taken up by T cells, inhibiting immune activation and the secretion of effectors such as TNF-α and IFN-γ, thus suppressing antitumour immunity and promoting GC progression.

In recent years, many studies have confirmed that circRNAs play biological roles through mechanisms such as competing endogenous RNAs (ceRNAs) or binding to RNA-binding proteins (RBPs). The ceRNA mechanism whereby circRNAs can competitively adsorb the corresponding microRNAs so that miRNAs are unable to bind with target genes and thus participate in the regulation of target gene expression. In the ceRNA model, circRNAs were shown to interact with the AGO2 protein [46, 47]. However, in our study, circMAN1A2 did not bind to AGO2 in RNA pull-down experiments. Therefore, we suggest that circMAN1A2 does not act through a ceRNA mechanism to play a role in GC progression. Consequently, we aimed to determine whether circMAN1A2 functions via the RBP mechanism. We performed mass spectrometry analysis of the RNA pull-down products of circMAN1A2 in HGC27 and Jurkat cells. SFPQ proteins were detected in the RNA pull-down products of both the HGC27 and Jurkat cell groups. This finding implies that circMAN1A2 functions by binding to SFPQ. Unexpectedly, we found that SFPQ was significantly negatively correlated with CD8 + T-cell infiltration in GC, according to the predictions of the TIMER 2.0 website.

Splicing factor proline- and glutamine-rich (SFPQ) proteins are multifunctional DNA- and RNA-binding proteins involved in various biological processes, such as RNA splicing, translocation, DNA repair, transcriptional regulation, and immune regulation. SFPQ is expressed in both the cytoplasm and nucleus, and aberrant SFPQ expression is potentially associated with many diseases, such as neurological disorders and malignancies. For example, SFPQ interacts with RNA to promote the expression of oncogenic transcripts in melanoma [48]. In colorectal cancer, SFPQ promotes tumour growth [49]. Therefore, we investigated the role of SFPQ in GC. By knocking down SFPQ expression, we found that GC cell migration and proliferation were inhibited. In the same case, in Jurkat cells, the secretion of antitumour immune-related effectors was elevated. These findings verified that SFPQ may be a downstream molecule through which circMAN1A2 functions.

We further analysed the single-cell data via the GEO public database. Following pathway enrichment analysis of differential SFPQ expression in epithelial cell and T-cell subpopulations of GC tissues. In epithelial cell subpopulations, SFPQ was able to regulate the cell cycle phase transition. In contrast, in T-cell subpopulations, SFPQ was able to affect the TCR signalling pathway. Combined with our previous analysis of the influence of circMAN1A2 on the cell cycle of GC cells, we found that changes in the cell cycle were mainly concentrated in the G1 and S phases. During the G1/S phase transition, Cyclin D1 (CCND1) is activated and binds cyclin-dependent kinase 4/6 (CDK4/6), which phosphorylates retinoblastoma protein (Rb). The E2F transcription factor is subsequently released, promoting cell cycle progression from the G1 phase to the S phase. Thus, we examined the expression levels of these important proteins in the G1/S phase transition under the influence of SFPQ via Western blot. The results showed that SFPQ was able to catalyse the G1/S phase transition and promote GC cell proliferation. The TCR signalling pathway has recently garnered increasing attention in the field of tumour immunity because it activates many signal transduction cascade reactions that ultimately determine the fate of target cells by regulating cytokine products, cell proliferation and differentiation. The key nodes of the TCR signalling pathway include mainly LCK, ZAP70, and LAT. The phosphorylation state of these essential signalling proteins is crucial in TCR signalling pathway activation [50, 51]. Thus, we examined the changes in these key signalling proteins and their phosphorylation levels. These results suggested that SFPQ affects T-cell activation and antitumour activity by inhibiting the TCR signalling pathway. We subsequently constructed a huPBMC-NCG mouse model and introduced the TCR signalling pathway agonist LYP-IN-3 in subcutaneous tumour experiments and then chose to overexpress circMAN1A2 in tumour cells. These results further confirmed that circMAN1A2 inhibits the activation of the TCR signalling pathway by binding to SFPQ to perform its biological function.

In the next mechanistic study, we found that circMAN1A2 inhibits ubiquitin–proteasome-mediated SFPQ protein degradation. Specifically, circMAN1A2 competes with FBXW11 for binding to SFPQ, preventing FBXW11-mediated k48-linked ubiquitination and SFPQ protein degradation. The ubiquitin**–**proteasome system (UPS) is an important component responsible for regulating protein homeostasis in cells [52]. Abnormalities in the UPS affect a variety of biological functions, such as protein localization, cell cycle progression, and apoptosis [53, 54], and are associated with the development of a variety of diseases, including malignancies [55]. UPS-mediated protein degradation is triggered by the ubiquitin-activating enzyme E1, which transfers ubiquitin to the ubiquitin-conjugating enzyme E2. Then, the E3 ubiquitin ligase attaches ubiquitin to the protein substrate. Among them, the diversity of E3 ligases is correlated with the specificity of the ubiquitination of various proteins in the cell, which makes E3 ligases play crucial roles in the UPS [56]. It has been reported that in GC, aberrant regulation of E3 ligases is associated with disease progression [57, 58].

FBXW11, known as F-box and WD-40 domain protein 11, is an important component of the SCF (Skp1-Cul1-F-box) E3 ubiquitin ligase complex. Among them, F-box proteins are the substrate recognition subunits of the SCF E3 ubiquitin ligase complex and play important roles in a variety of cellular processes through the ubiquitination of target proteins and subsequent protein degradation [59]. The F-box family can be classified into the FBXW, FBXL and FXBO subfamilies [60]. Related studies have reported that F-box-mediated protein homeostasis dysregulation plays a critical role in a variety of malignant tumours [59, 61, 62]. Several studies have reported the role of FBXW11 in malignancies. Yao et al. reported that FBXW11 promotes liver metastasis in colorectal cancer by regulating HIC1-mediated SIRT1 expression [34]. Tan et al. reported that FBXW11 was associated with immune infiltration and NF-κB pathway activation in pancreatic cancer [35]. Our study is the first to report the role of FBXW11 in GC. FBXW11 acts as an E3 ubiquitin ligase for SFPQ and mediates SFPQ protein degradation. circMAN1A2 competes with FBXW11 for binding to SFPQ, thereby inhibiting SFPQ degradation and promoting GC progression and immunosuppression.

There may be limitations to our findings. In our study, we mainly focused on the ubiquitination process, revealing the mechanism of circMAN1A2 inhibiting FBXW11-mediated SFPQ degradation. However, circRNAs have also been reported to be implicated in regulating protein deubiquitination. For example, Zhuang et al. found that circ-0100519 enhances the interaction between the deubiquitinating enzyme ubiquitin-specific protease 7 (USP7) and nuclear factor-like 2 (NRF2) in macrophages, promoting USP7-mediated deubiquitination of NRF2, which induces M2 macrophage polarization and promotes breast cancer progression [63]. Similarly, Yang et al. reported that hypoxia-induced circWSB1 interacts with USP10, reducing USP10-mediated stabilization of p53, leading to p53 degradation and promoting breast cancer progression [64]. Ubiquitination and deubiquitination is a complex dynamic equilibrium mechanism [65, 66], and there may be additional deubiquitination mechanisms not explored in our study. Further research is needed to better understand the molecular mechanisms regulating GC progression.

In summary, our study reveals the biological role of circMAN1A2 in promoting GC progression; moreover, circMAN1A2 can be delivered to T cells in the form of exosomes to promote immunosuppression. Our analysis of the clinical data suggests that circMAN1A2 has potential clinical applications in both the diagnosis and prognosis of GC, providing new insights for subsequent guidance of systemic therapy for GC.

## Conclusions

**Fig. 10:**
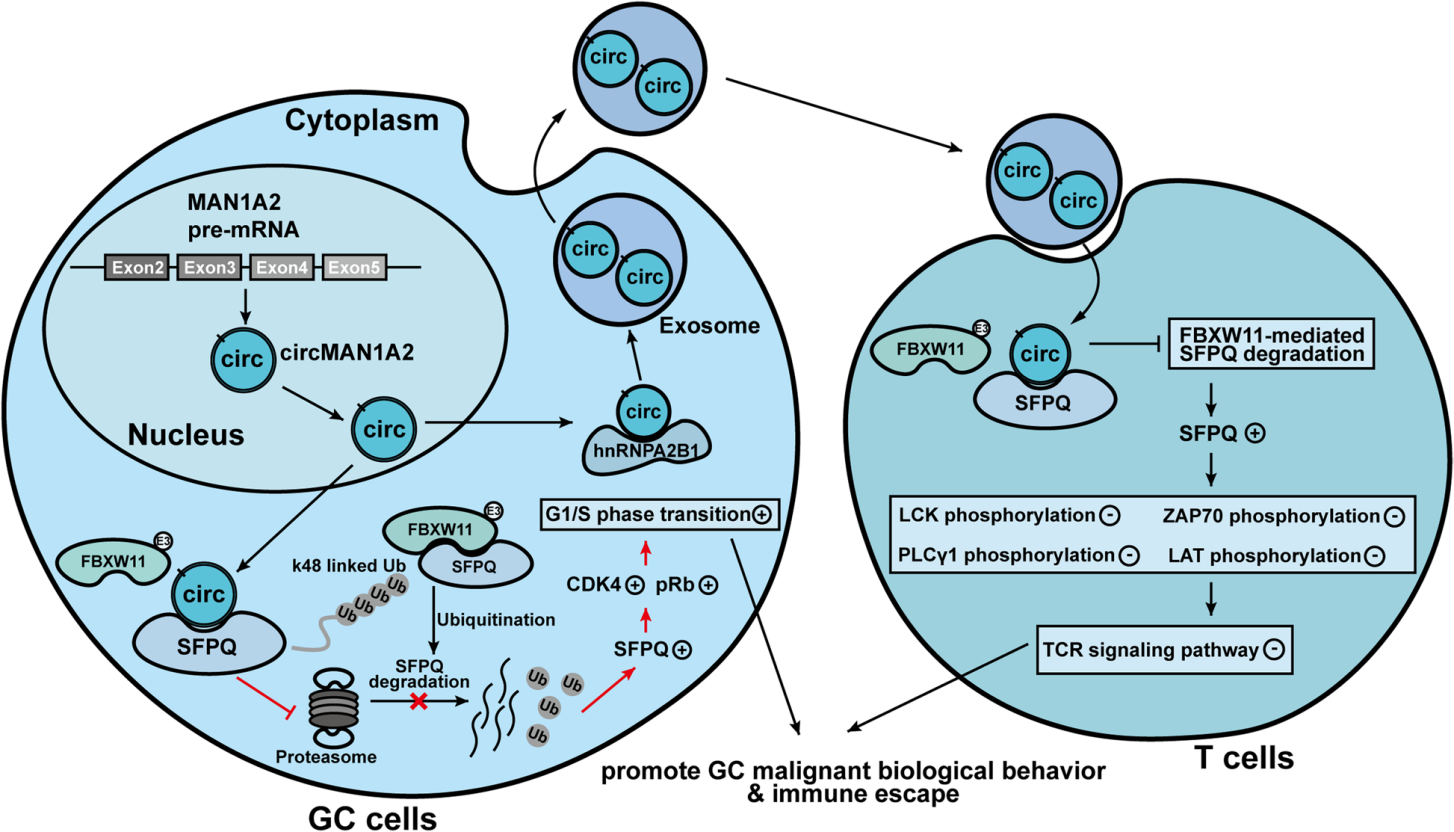
Schematic model showing the mechanism and role of exosomal circMAN1A2 in gastric cancer

In conclusion, our work revealed the upregulation of circMAN1A2 in GC-derived exosomes, which is associated with the diagnosis and prognosis of GC patients. circMAN1A2 can be encapsulated by hnRNPA2B1 in exosomes and can be taken up by T cells, thus affecting antitumour immunity. Mechanistically, circMAN1A2 plays a role in promoting GC development by interacting with SFPQ in GC cells and T cells, affecting the G1/S phase transition of the cell cycle in GC cells while inhibiting TCR signalling pathway activation in T cells. circMAN1A2 is able to competitively bind to SFPQ with FBXW11, preventing FBXW11-mediated k48-linked ubiquitination and SFPQ protein degradation, thereby stabilizing SFPQ expression (Fig. 10).

The role of circMAN1A2 in GC progression and antitumour immunity provides a theoretical basis for it to be a potential therapeutic target for GC, and also provides new insights into the future development of immunotherapy for GC. More exosomal circRNA biomarkers may be investigated to help understand more comprehensively the role of exosomal circRNAs in GC complex biological processes and to promote clinical translation of the basic research. In the near future, we hoped circMAN1A2-based neoantigen vaccines can be developed in preventing and treating GC.

## Supplementary Information

Supplementary Material 1.

Supplementary Material 2.

Supplementary Material 3: Sup Fig. 1 A. The start-end length distributions of the genes corresponding to the 33,997 circRNAs. B. The chromosomal locus distributions of these circRNAs. C. After obtaining the intersection of three groups, 75 circRNAs enriched in GC-derived exosomes were identified. D. The chromosomal locus distributions of the 75 GC-derived exosomal circRNAs. E&F. Relative expression of the 9 circRNAs at the transcriptional level in 40 pairs of GC and adjacent normal tissues from GC patients. G. Relative expression of hsa_circ_0000118 in exosomes derived from 20 pairs of GC and adjacent normal tissues from GC patients.

Supplementary Material 4: Sup Fig. 2 A. The expression levels of circMAN1A2 in six different human GC cell lines (HGC27, MKN28, KATOIII, SNU1, MKN45, and AGS). Data were normalized to the expression levels of circMAN1A2 in normal gastric mucosal tissue. B. Relative levels of circMAN1A2 and MAN1A2 mRNA were measured by qRT-PCR in AGS treated with Actinomycin D for different periods of time. C. Relative levels of GAPDH (positive control for cytoplasmic fraction), U6 (positive control for nuclear fraction), circMAN1A2, and MAN1A2 mRNA from cytoplasmic and nuclear fractions in AGS. Graph represents mean ± SD; *p < 0.05, **p < 0.01, and ***p < 0.001.

Supplementary Material 5: Sup Fig. 3 A. Efficiency verification of three small interfering RNAs (si-RNAs) by qRT-PCR in HGC27 cells. B. Efficiency verification of three small interfering RNAs (si-RNAs) by qRT-PCR in AGS cells. C. Efficiency verification of circMAN1A2 overexpression plasmid by qRT-PCR in HGC27 and AGS cells. Graph represents mean ± SD; *p < 0.05, **p < 0.01, and ***p < 0.001.

Supplementary Material 6: Sup Fig. 4 A&B. The colony formation assay was performed to evaluate proliferation ability after upregulating or downregulating circMAN1A2 in AGS cells. C-E. The CCK8 assay was performed to evaluate proliferation ability after upregulating or downregulating circMAN1A2 in AGS cells. F&G. The EdU incorporation assay was performed to evaluate proliferation ability after upregulating or downregulating circMAN1A2 in AGS cells. Scale bar: 100 μm. H&I. The wound healing experiment was performed to evaluate migration ability after upregulating or downregulating circMAN1A2 in AGS cells. Scale bar: 500 μm. J&K. Transwell assay was performed to evaluate migration ability after upregulating or downregulating circMAN1A2 in AGS cells. Scale bar: 100 μm. L&M. The effect of circMAN1A2 on modulating AGS cell cycle progression was evaluated by flow cytometry assay. Graph represents mean ± SD; *p < 0.05, **p < 0.01, and ***p < 0.001.

Supplementary Material 7: Sup Fig. 5 A. Relative expression of circMAN1A2 in HGC27-derived exosomes from different treatment groups. B&C. The colony formation assay was performed to evaluate HGC27 cell proliferation ability after cocultured with exosomes from HGC27 cells upregulating or downregulating circMAN1A2. D. The CCK8 assay was performed to evaluate HGC27 cell proliferation ability after cocultured with exosomes from HGC27 cells upregulating or downregulating circMAN1A2. E&F. The EdU incorporation assay was performed to evaluate HGC27 cell proliferation ability after cocultured with exosomes from HGC27 cells upregulating or downregulating circMAN1A2. Scale bar: 100 μm. G&H. The wound healing experiment was performed to evaluate HGC27 cell migration ability after cocultured with exosomes from HGC27 cells upregulating or downregulating circMAN1A2. Scale bar: 500 μm. I&J. Transwell assay was performed to evaluate HGC27 cell migration ability after cocultured with exosomes from HGC27 cells upregulating or downregulating circMAN1A2. Scale bar: 100 μm. Graph represents mean ± SD; *p < 0.05, **p < 0.01, and ***p < 0.001.

Supplementary Material 8: Sup Fig. 6 A. Immunohistochemistry staining of E-cadherin in respective xenograft tumour tissues. Scale bar: 50 μm. B. IHC scores of E-cadherin in respective xenograft tumour tissues. Graph represents mean ± SD; *p < 0.05, **p < 0.01, and ***p < 0.001.

Supplementary Material 9: Sup Fig. 7 A. Protein bands detected by silver stain for mass spectrometry of the circMAN1A2-protein complex pulled down by sense or anti-sense circMAN1A2 in HGC27 cells. B. The typical hnRNPA2B1 peptide was identified in circMAN1A2-enriched proteins based on MS analysis. C. RIP and qRT-PCR assays showed the interaction between hnRNPA2B1 and circMAN1A2 in HGC27 and AGS cells, using IgG and hnRNPA2B1 antibodies, *n*=3. D. RNA pull-down and Western blot assays were performed to confirm the interaction between hnRNPA2B1 and circMAN1A2 in HGC27 and AGS cells. E. qRT-PCR assays showed the relative expression levels of circMAN1A2 in HGC27 and AGS cells transfected with si-NC or si-hnRNPA2B1, and in exosomes extracted from HGC27 and AGS cells transfected with si-NC or si-hnRNPA2B1, *n*=3. Graph represents mean ± SD; *p < 0.05, **p < 0.01, and ***p < 0.001.

Supplementary Material 10: Sup Fig. 8 A. RNA pull-down experiments were performed in HGC27 and Jurkat cells to detect AGO2 protein. B. Correlation of SFPQ with CD8+ T cell infiltration in GC. C. RPIseq prediction revealed the binding potential of SFPQ with circMAN1A2.

Supplementary Material 11: Sup Fig. 9 A. Efficiency verification of si-SFPQ in HEK293T cells. B. Statistical graphs of EdU positive cells in HGC27 and AGS cells. C. Statistical graphs of the colony number in HGC27 and AGS cells. D. Statistical graphs of the migrated cell number in HGC27 and AGS cells. E. Statistical graphs of the S period proportion in the cell cycle process in HGC27 and AGS cells. F. Statistical graphs of CD8+ IFN-γ+ Jurkat cells. G. Statistical graphs of CD8+ TNF-α+ Jurkat cells. H. Statistical graphs of HGC27 apoptosis rates. I. Statistical graphs of TUNEL positive cells in HGC27. Graph represents mean ± SD; *p < 0.05, **p < 0.01, and ***p < 0.001.

Supplementary Material 12: Sup Fig. 10 A. The predicted regions in circMAN1A2 which exhibit the potential to bind to SFPQ. B. Different circMAN1A2 truncated probes were designed based on the potential binding regions. C. RNA pull-down and Western blot assays were performed to identify the regions binding to SFPQ.

Supplementary Material 13: Sup Fig. 11 A. Co-IP experiment was performed in HEK293T cells using SFPQ antibody. B. The typical FBXW11 peptide was identified in co-IP protein samples based on MS analysis. C. Co-IP assays confirmed that silencing circMAN1A2 increases the binding between FBXW11 and SFPQ.

## Data Availability

All data in the current study are available from the corresponding author upon reasonable request.

## Abbreviations

GC: Gastric cancer
circRNA: Circular RNA
EV: Extracellular vesicle
TME: Tumour microenvironment
CCK-8: Cell counting Kit-8
qRT-PCR: Quantitative real-time polymerase chain reaction
EdU: 5-Ethynyl-2′-deoxyuridine
FISH: Fluorescence in situ hybridization
IF: Immunofluorescence
co-IP: Co-immunoprecipitation
RIP: RNA immunoprecipitation
IHC: Immunohistochemistry
mIHC: Multiplex immunohistochemistry
pre-mRNA: Precursor mRNA
NTA: Anoparticle tracking analysis
cDNA: Complementary DNA
gDNA: Genomic DNA
ROC: Receiver operating characteristic
siRNAs: Small interfering RNAs
MS: Mass spectrometry
LM: Liver metastasis
ceRNAs: Competing endogenous RNAs
RBPs: RNA-binding proteins
SFPQ: Splicing factor proline- and glutamine-rich
DEGs: Differentially expressed genes
TCR: T-cell receptor
CCND1: Cyclin D1
CDK4/6: Cyclin-dependent kinase 4/6
pRb: Phosphorylated retinoblastoma
PBMCs: Peripheral blood mononuclear cells
RRM: RNA recognition motif
CCR: Coiled coil region
UPS: Ubiquitin–proteasome system
CTLs: Cytotoxic T lymphocytes
FBXW11: F-box and WD repeat domain containing 11
SCF: SKP1-CUL1-F-box
