# Supplementary material for: GC-derived exosomal circMAN1A2 promotes cancer progression and suppresses T-cell antitumour immunity by inhibiting FBXW11-mediated SFPQ degradation
Source: J Exp Clin Cancer Res. 2025 Jan 25;44:24. doi: 10.1186/s13046-025-03288-9 (PMC11762487; doi:10.1186/s13046-025-03288-9)
Supplement: Supplementary file 1 — Supplementary Material 1. [file 13046_2025_3288_MOESM1_ESM.docx]

**Antibodies used in this study**

| **Antibodies** | **Source** | **Catalog** | **Application** |
| --- | --- | --- | --- |
| anti-Calnexin | abcam | ab22595 | WB |
| anti-TSG101 | abcam | ab133586 | WB |
| anti-CD81 | abcam | ab109201 | WB |
| anti-Alix | abcam | ab275377 | WB |
| anti-CD9 | abcam | ab236630 | WB |
| anti-hnRNPA2B1 | abcam | ab31645 | WB, IP |
| anti-GAPDH | abcam | ab8245 | WB |
| anti-AGO2 | abcam | ab186733 | WB |
| anti-CDK4 | Cell Signaling Technology | 12790 | WB |
| anti-CDK6 | Cell Signaling Technology | 30483 | WB |
| anti-CCND1 | Cell Signaling Technology | 55506 | WB |
| anti-pRb | Cell Signaling Technology | 8516 | WB |
| anti-Phospho-Zap-70 (Tyr319) | Cell Signaling Technology | 2717 | WB |
| anti-Zap-70 | Cell Signaling Technology | 2705 | WB |
| anti-Phospho-PLCγ1 (Tyr783) | Cell Signaling Technology | 14008 | WB |
| anti-PLCγ1 | Cell Signaling Technology | 2822 | WB |
| anti-Phospho-LCK (Tyr394) | Cell Signaling Technology | 70926 | WB |
| anti-Phospho-LAT (Tyr220) | Cell Signaling Technology | 3584 | WB |
| anti-LAT | Cell Signaling Technology | 45533 | WB |
| anti-SFPQ | abcam | ab11825 | WB, IP, IF |
| anti-HA tag | abcam | ab9110 | WB |
| anti-flag tag | Cell Signaling Technology | 14793 | WB, IP |
| anti-myc tag | abcam | ab9106 | WB |
| anti-Ki67 | abcam | ab15580 | IHC |
| anti-E-cadherin | abcam | ab40772 | IHC |
| FITC anti-human CD3 Antibody | BioLegend | 317306 | Flow |
| PerCP/Cyanine5.5 anti-human CD8 Antibody | BioLegend | 344710 | Flow |
| PE anti-human IFN-γ Antibody | BioLegend | 383304 | Flow |
| PE anti-human TNF-α Antibody | BioLegend | 502909 | Flow |
| anti-CD8 | abcam | ab237709 | mIHC |
| anti-GZMB | abcam | ab208586 | mIHC |

**Primers used in this study**

| Primer | | Sequence (5′-3′) |
| --- | --- | --- |
| circMAN1A2  (divergent primers) | Forward | ATTGGAGGCCTACTTGCAGC |
|  | Reverse | GGGTCTCCACCACGTATTCC |
| circMAN1A2  (convergent primers) | Forward | GGAATACGTGGTGGAGACCC |
|  | Reverse | GGGGAGTGTCCTTTCCTTGC |
| GAPDH | Forward | AATGGGCAGCCGTTAGGAAA |
|  | Reverse | GCGCCCAATACGACCAAATC |
| circ0013738 | Forward | CTTCCTGCCTTTAACACACC |
|  | Reverse | TCAATCCATCTTTGCCCATC |
| circ0000117 | Forward | GGCAAAGATGGATTGAAGAC |
|  | Reverse | GTTGGGAATAGGGACTGGTG |
| circ0013734 | Forward | GCAGCATATTACCTATCAGG |
|  | Reverse | TAAAGGGTATCCAAAGCATC |
| circ0000119 | Forward | GGATTCCTTGGGCAATGGTG |
|  | Reverse | TGGTGGCAGTGGCTTGTTCT |
| circ0000116 | Forward | TGCCACCAGTCCCTATTCCC |
|  | Reverse | CGTTCTTCCTCTTCCCAAAT |
| circ0065251 | Forward | TCTTGCCTCAGCGGCTACCA |
|  | Reverse | TTATCACCTTCATCCGTTTC |
| circ0005780 | Forward | ATGAGTCTCGAAGTGAAATT |
|  | Reverse | GTGAGGAAGATGAGTGATGT |
| circ0019079 | Forward | AGAGTAATGAAATGGAGGAG |
|  | Reverse | TCTTCTAGTGTCATTCGCAT |
| MAN1A2 | Forward | CACTGCCACCAGTCCCTATT |
|  | Reverse | TCTTCAATCCATCTTTGCCC |
| hnRNPA2B1 | Forward | CAGCAACCTTCTAACTACGGTCC |
|  | Reverse | CACTGCCTCCTGGACCATAGTT |
| SFPQ | Forward | AGCGTCTTCTTCGCTTTTGC |
|  | Reverse | GAGAACGGAAGTCGTGGAGG |
| FBXW11 | Forward | GTGGGATGTGAACACGGGTGA |
|  | Reverse | CGTAAAGTGATGTCGGTCGCAG |

**DNA/RNA sequence used in this study**

| si-RNA | Sequence |
| --- | --- |
| si-NC-1 | 5’-AGAAGGCUCCUCUCCUGAUAGTT-3’ |
| si-circMAN1A2-1 | 5’-UCUUCCCUCCUCUCCUGAUAGTT-3’ |
| si-NC-2 | 5’-UUCUUCCUCUUCCGAGGAGAGTT-3’ |
| si-circMAN1A2-2 | 5’-UUCUUCCUCUUCCCUCCUCUCTT-3’ |
| si-NC-3 | 5’-AAGGAGAAGGCUCCUCUCCUGTT-3’ |
| si-circMAN1A2-3 | 5’-UUCCUCUUCCCUCCUCUCCUGTT-3’ |
| si-hnRNPA2B1-1 | 5’-AUUGAUGGUAUGGUAUUUCUGTT-3’ |
| si-hnRNPA2B1-2 | 5’-UAAGCCACCAAUAAAGAGCTT-3’ |
| si-SFPQ-1 | 5’-UAGAUUCCCAACAAACAACCGTT-3’ |
| si-SPFQ-2 | 5’-UAACUUUCCUCUCUUUGGCGCTT-3’ |
| si-NC | 5’-UUCUCCGAACGUGUCACGUTT-3’ |
