# Supplementary material for: GC-derived exosomal circMAN1A2 promotes cancer progression and suppresses T-cell antitumour immunity by inhibiting FBXW11-mediated SFPQ degradation
Source: J Exp Clin Cancer Res. 2025 Jan 25;44:24. doi: 10.1186/s13046-025-03288-9 (PMC11762487; doi:10.1186/s13046-025-03288-9)
Supplement: Supplementary file 2 — Supplementary Material 2. [file 13046_2025_3288_MOESM2_ESM.docx]

**Materials and Methods**

**Exosomes and Jurkat cells co-culture system**

Exosomes derived from HGC27 and AGS cells were isolated as described previously. Jurkat cells were pre-activated for 24 h with 2μg/mL of soluble human CD3/CD28 T cell activator (Proteintech, China). Jurkat cells were then mixed with 100μg/mL of exosomes. After co-incubation for 72 hours, cells were collected for Western blot.

**Actinomycin D and RNase R treatment**

HGC27 and AGS cells were transferred to 24-well plates overnight, then treated with 2 μg/mL actinomycin D and collected at different pre-set time points (0, 6, 12 ,18, and 24 h). RNA was then extracted. The levels of circMAN1A2 and MAN1A2 mRNA were assessed by qRT-PCR.

For RNase R digestion, total RNA (5 μg) was incubated with or without 5 U/μg RNase R (Geneseed Biotech, China) at 37℃ for 15 min. The reverse transcription was performed and the remaining levels of circMAN1A2 and MAN1A2 mRNA were calculated by qRT-PCR.

**Plasmid, siRNA, lentiviral construction, and cell transfection**

The human circMAN1A2 plasmids and si-circMAN1A2 were purchased from GeneChem (Shanghai, China) to establish circMAN1A2 overexpression or knockdown GC cell lines. The transfection process was carried out following the manufacturer’s protocol of Lipofectamine 3000 (Invitrogen, USA). The lentiviruses were constructed by GeneChem (Shanghai, China) for stable transfection.

**Cell counting Kit-8 (CCK-8) assays**

Cells were seeded in 96-well plates at a density of 1,500 cells per well. Following the manufacturer’s protocol, the proliferation rates of GC cells were calculated with the Cell Counting Kit-8 reagent (Dojindo, Japan). For each well, the optical density (OD) values at 450 nm were acquired every 24h for 5 times.

**Colony formation assays**

The treated GC cells were seeded in 6-well plates (500 cells per well) and cultured in complete medium for two weeks. Then, the colonies were fixed with 2 mL of paraformaldehyde for 30 min and stained with 1% crystal violet for 20 min at room temperature. Finally, we counted the number of colonies.

**5-Ethynyl-2′-deoxyuridine (EdU) incorporation assays**

The EdU assay was carried out with an EdU cell proliferation assay kit (Beyotime, China) to detect cell proliferation. 20,000 GC cells were seeded into 96-well plates and incubated with EdU (10μM) for 2h. Then, the cells were treated with 4% paraformaldehyde for 20 min and permeabilized with 0.3% Triton X-100 for 10 min. Click Additive Solution (50 μL) was added to stain EdU for 30 min, then Hoechst 33342 was employed to stain nucleus for 10 min. Finally, images were acquired using a inverted fluorescence microscope (Nikon, Japan), and the cell proliferation rate was calculated by the ratio of EdU-positive cell count to Hoechst-positive cell count.

**Wound healing assays**

8×10^5^ GC cells were seeded into 6-well plates, when cells overgrew the bottom of the plate, a 200 μL pipette tip was used to create scratches in each well. After removing cellular fragments with PBS, each wound was recorded at 0 h with an inverted microscope (Olympus, Japan). Then, cells were continually cultured in basal medium. When it came to 48 h, the wounds were imaged after the same washing procedure aforementioned. Cell migration was assessed via ImageJ.

**Transwell assays**

Transwell inserts (Corning, USA) were employed to assess the migration ability of GC cells. 24-well plates were used to load chambers. 800 μL complete medium was added to the bottom of each well, then Transwell inserts were put on the well and 200 μL serum-free medium together with 2×10^4^ GC cells were added in the upper chamber. After 48 h, the culture medium was removed and the cells attached to the membrane were fixed with paraformaldehyde and stained with 1% crystal violet. The upper chamber was wiped with cotton and the cells adhering to the bottom chamber were then observed through microscope.

**Flow cytometry**

For cell cycle assay, 1×10^6^ cells were collected. After centrifugation, the supernatant was discarded and the cell precipitate was washed by PBS. Centrifuge again and remove the supernatant. Then 1 mL DNA staining solution together with 10 μL Permeabilization solution were mixed into the cell precipitate. After blowing evenly, incubate for 30 min according to the protocol of Cell Cycle Staining Kit (MultiSciences, China). The cell distribution was measured by flow cytometry.

For apoptosis analysis, Annexin V-FITC Apoptosis Detection Kit I (BD Biosciences, USA) was employed to stain cells according to the protocol. The levels of cell apoptosis were measured by flow cytometry.

For T-cell effector assay, anti-IFN-γ-FITC (BioLegend, USA), anti-TNF-α-PE (BioLegend, USA) and anti-CD8-PerCP (BioLegend, USA) were employed to detect CD8^+^IFN-γ^+^ or CD8^+^TNF-α^+^ cells.

**RNA fluorescence in situ hybridization (FISH)**

Cells were fixed in 4% paraformaldehyde for 20 min, washed with PBS and permeabilized with 0.1% Triton X-100. Hybridization of cells were performed with Cy3-labeled circMAN1A2 probes in a damp dark chamber at 37 ℃ overnight. Before imaging, the slides were cleaned, dried, and dyed with DAPI. Servicebio (Wuhan, China) designed and produced the RNA FISH probe. A Leica SP5 confocal microscope equipment (Leica Microsystems, Germany) was used to capture the images.

**Immunofuorescence (IF)**

To further assess the co-localization of circMAN1A2 and SFPQ, HGC27 and Jurkat cells were transfected with Cy3-labeled circMAN1A2 probes and cultured with anti-SFPQ antibody. DAPI was used to stain the nuclei of cells. Finally, the Leica SP5 confocal microscope equipment (Leica Microsystems, Germany) was used to capture the images.

**Co-immunoprecipitation (co-IP)**

Co-IP was performed with Pierce Classic Magnetic IP/Co-IP Kit (Thermo Fisher Scientific, USA) following the manufacturer's protocol. Briefly, Cells were lysed in 500μL Pierce IP Lysis. The lysates were centrifuged for 10 min at 13,000 ×g. Then, the supernatant was incubated with antibodies for 12 h at 4 °C. The antigen-antibody complex was bound to Protein A/G magnetic beads for 2 hours at room temperature. The beads were washed twice with Wash Buffer and once with purified water. Finally, the antigen-antibody complex was eluted and samples were analyzed by Western blot and mass spectrometry.

**RNA immunoprecipitation (RIP)**

The RNA-Binding Protein Immunoprecipitation Kit (Millipore, USA) was employed to carry out RIP assay according to the manufacturer’s protocol. Briefly, approximately 2×10^7^ cells were lysed in 100 μL RIP lysis buffer (containing 0.5 μL of protease inhibitor cocktail and 0.25 μL of RNase inhibitor) on ice for 5 min. The supernatant was collected by centrifugation. A portion of supernatant was as the input. The other portion of supernatant was added 50 µL beads complex with 5 µg antibody against target protein or corresponding IgG, and incubated overnight at 4 ℃. Next, the immunoprecipitation tubes was centrifuged briefly and placed on the magnetic separator. The supernatant was discarded. Then, 500μL of RIP Wash Buffer was added to each tube. The tubes were placed on the magnetic separator again to discard the supernatant. Totally, the washing step was repeated fore six times. The protein was then digested and the RNA was purified by the solution of phenol:chloroform:isoamyl alcohol. NanoDrop ND-2000 spectrophotometer (Thermo Fisher Scientific, USA) was used to assess the concentration and quality of the extracted RNA. Reverse-transcription and qRT-PCR procedures were performed as described previously to identify and analyze the captured RNA.

**RNA pull-down assays**

Biotinylated circMAN1A2 and its anti-sense sequence were synthesized by RiboBio (GenePharma, China). Cells were lysed and incubated with a biotin-labelled circMAN1A2 probe. Then, cell lysates were incubated with streptavidin magnetic beads (Thermo Fisher Scientific, USA) at room temperature. The beads were collected with a magnetic stand, and then washed with Binding/Wash Buffer for 3 times. Finally, interacting proteins were identifed by Western blot and mass spectrometry.

**Immunohistochemistry (IHC) staining**

The collected tissues were incubated with 4% paraformaldehyde immediately after removing from the living body, then embedded in paraffin, cut into sections. 3% hydrogen peroxide was added to suppress endogenous peroxidase activity for 10 min. Then the sections were incubated with primary antibody at 4℃ overnight. After two PBS washes the next day, sections were incubated with the HRP polymer-conjugated secondary antibody for 1 h at room temperature (Abcam, UK). Slices were then stained by 3,3-diaminobenzidine solution and haematoxylin. At last, slices were observed with a microscope (Olympus, Japan), and the pictures were taken for further analysis.

**Animal study**

The animal experiments involved in this study were approved by the Animal Ethics Committee of Nanjing Medical University. Four-week-old BALB/c nude mice were purchased from the Laboratory Animal Centre of Nanjing Medical University. The huPBMC-NCG mouse model was constructed by Gempharmatech. The mice were grouped according to the pre-designed experimental protocol. To study the xenograft tumour growth, 1×10^6^ transfected cells were resuspended in 100 μL PBS and subcutaneously injected into axilla of nude mice or huPBMC-NCG mice. LYP-IN-3 (also known as compound D34, MedChemExpress, China) and Pembrolizumab (anti-PD-1, MedChemExpress, China) were employed to treat the huPBMC-NCG mice to investigate the relationship between circMAN1A2 and the TCR signaling pathway. LYP-IN-3 was dissolved with PBS containing 5% DMSO. The therapeutic dose was 50 mg/kg twice a day. For PD-L1 blockade, mice were given intraperitoneal injections of Pembrolizumab at a dose of 4 mg/kg once every two days. Both LYP-IN-3 and Pembrolizumab were started from the first week after cell injection. The length and width of the tumour were measured once a week and the volume of the tumour was thereby estimated. The calculation formula was (width^2^ × length)/ 2. After 4 weeks, the mice were sacrificed and the subcutaneous tumours were acquired. The volume and weight of the tumours in each group were measured.

For in vivo metastasis model analysis, we performed the construction of a liver metastasis model for GC. 1×10^6^ transfected luciferase-labeled GC cells were resuspended in 100 μL PBS and injected into the spleens of nude mice. Four weeks later, mice were injected intraperitoneally with D-luciferin sodium salt (Genomeditech, China) working solution (15 mg/ml) at a dose of 10 μL of working solution per gram of mice body weight. After anesthesia by inhalation of 2% isoflurane, metastasis in mice was monitored using the IVIS Spectrum Imaging System (IVIS; Caliper Life Sciences, USA). Subsequently, the livers were removed from mice for photography and H&E staining. Finally, H&E stained sections were quantitatively analyzed for sites of liver metastasis by a microscope.

**Single-cell RNA sequencing data processing**

We searched the GEO database (https://www.ncbi.nlm.nih.gov/geo/) for single-cell data related to gastric cancer using the keywords "gastric cancer" and "single cell", and finally selected two datasets (GSE183904, GSE150290) for subsequent analysis. First, the cell matrix file of each sample in the two datasets was imported into the R software separately with the CreateSeuratObject function in Seurat (version 5.0.3). Next, cells with more than 200 genes detected and a total transcript count over 200, but not surpassing 10,000 were selected. Cells with more than 20% of transcripts from mitochondrial genes were considered potentially apoptotic and thus excluded. Each dataset was integrated and corrected for batch effects using Harmony, and then the 2 single-cell datasets were further conducted integration and batch-effect correction. To identify major cell populations, we normalized the meta dataset using the NormalizeData function in Seurat. The top 2000 variable features were then selected based on the variance stabilizing transformation (VST). Subsequently, principal component analysis (PCA) was used for dimensionality reduction to identify cell clusters at a resolution of 0.5. Finally, the dimensionality of the cell clusters was reduced using UMAP. For the acquired data, cell clusters were identified using "singleR", from which epithelial cell and T cell subpopulations were selected for subsequent analysis, respectively. Then, we performed dimensionality reduction clustering of epithelial cell and T cell subpopulations using the same method, respectively, and visualized the distribution of SFPQ genes in each subpopulation with "FeaturePlot". Based on the expression level of SFPQ genes, we divided the two cell subpopulations into high-expression and low-expression groups, respectively, with 2 (epithelial cells) and 1 (T cells) as the boundary. Then we screened for differential genes using "FindMarkers", and then performed GO and KEGG enrichment analyses based on the differential genes.
