# Supplementary figures and images for: GC-derived exosomal circMAN1A2 promotes cancer progression and suppresses T-cell antitumour immunity by inhibiting FBXW11-mediated SFPQ degradation
Source: J Exp Clin Cancer Res. 2025 Jan 25;44:24. doi: 10.1186/s13046-025-03288-9 (PMC11762487; doi:10.1186/s13046-025-03288-9)

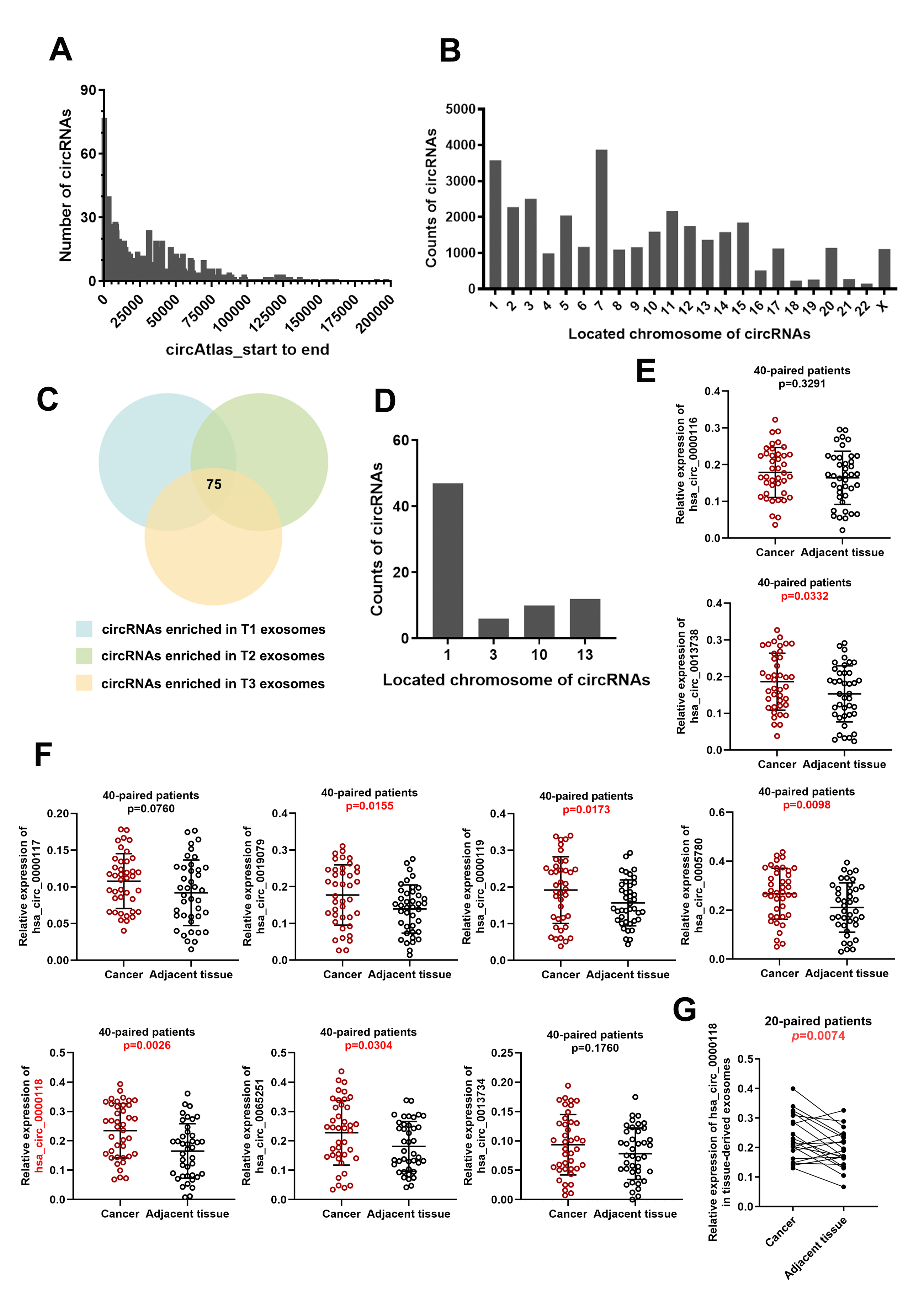

Supplement: Supplementary file 3 — Supplementary Material 3: Sup Fig. 1 A. The start-end length distributions of the genes corresponding to the 33,997 circRNAs. B. The chromosomal locus distributions of these circRNAs. C. After obtaining the intersection of three groups, 75 circRNAs enriched in GC-derived exosomes were identified. D. The chromosomal locus distributions of the 75 GC-derived exosomal circRNAs. E&F. Relative expression of the 9 circRNAs at the transcriptional level in 40 pairs of GC and adjacent normal tissues from GC patients. G. Relative expression of hsa_circ_0000118 in exosomes derived from 20 pairs of GC and adjacent normal tissues from GC patients. [file 13046_2025_3288_MOESM3_ESM.tif]

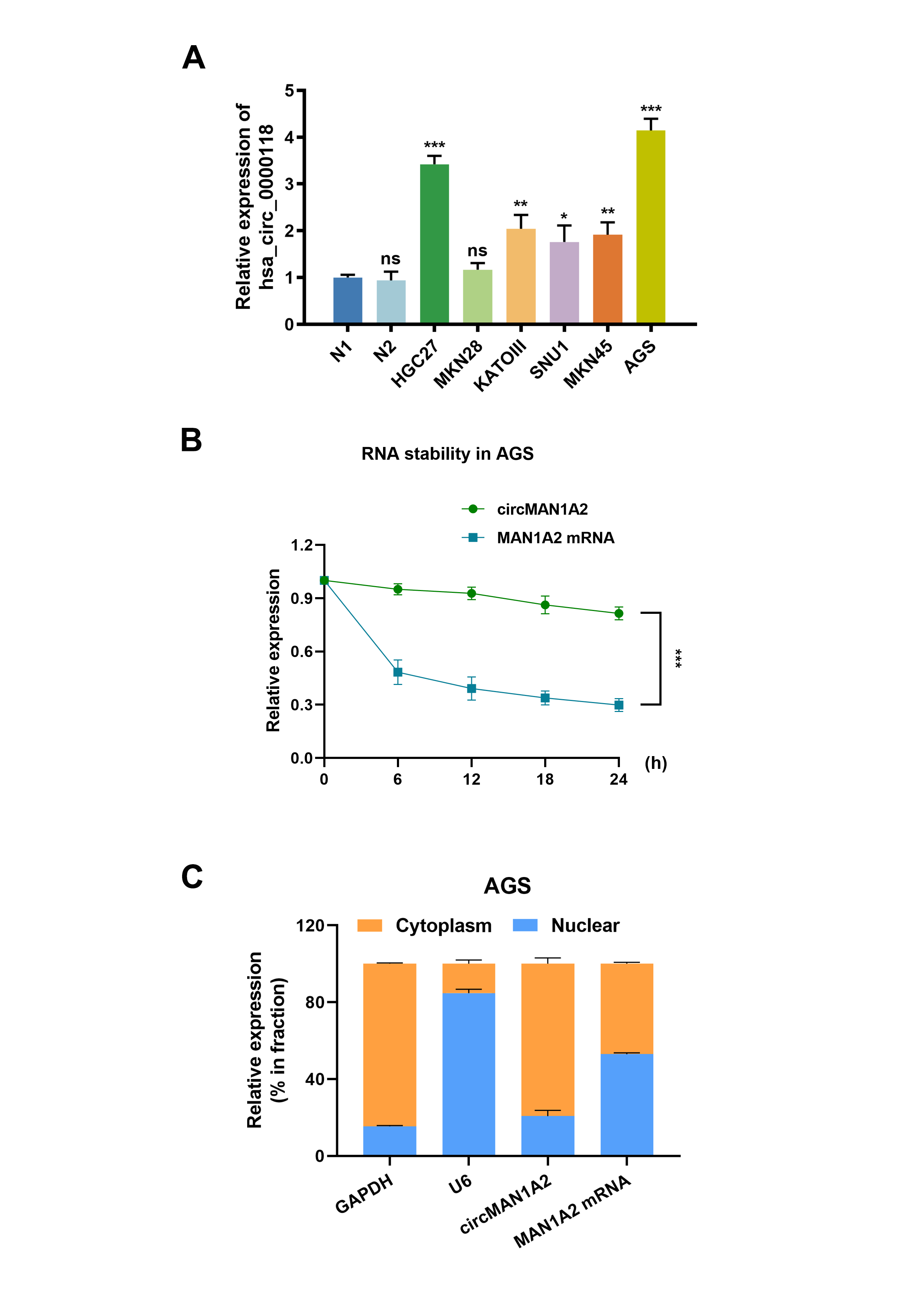

Supplement: Supplementary file 4 — Supplementary Material 4: Sup Fig. 2 A. The expression levels of circMAN1A2 in six different human GC cell lines (HGC27, MKN28, KATOIII, SNU1, MKN45, and AGS). Data were normalized to the expression levels of circMAN1A2 in normal gastric mucosal tissue. B. Relative levels of circMAN1A2 and MAN1A2 mRNA were measured by qRT-PCR in AGS treated with Actinomycin D for different periods of time. C. Relative levels of GAPDH (positive control for cytoplasmic fraction), U6 (positive control for nuclear fraction), circMAN1A2, and MAN1A2 mRNA from cytoplasmic and nuclear fractions in AGS. Graph represents mean ± SD; *p < 0.05, **p < 0.01, and ***p < 0.001. [file 13046_2025_3288_MOESM4_ESM.tif]

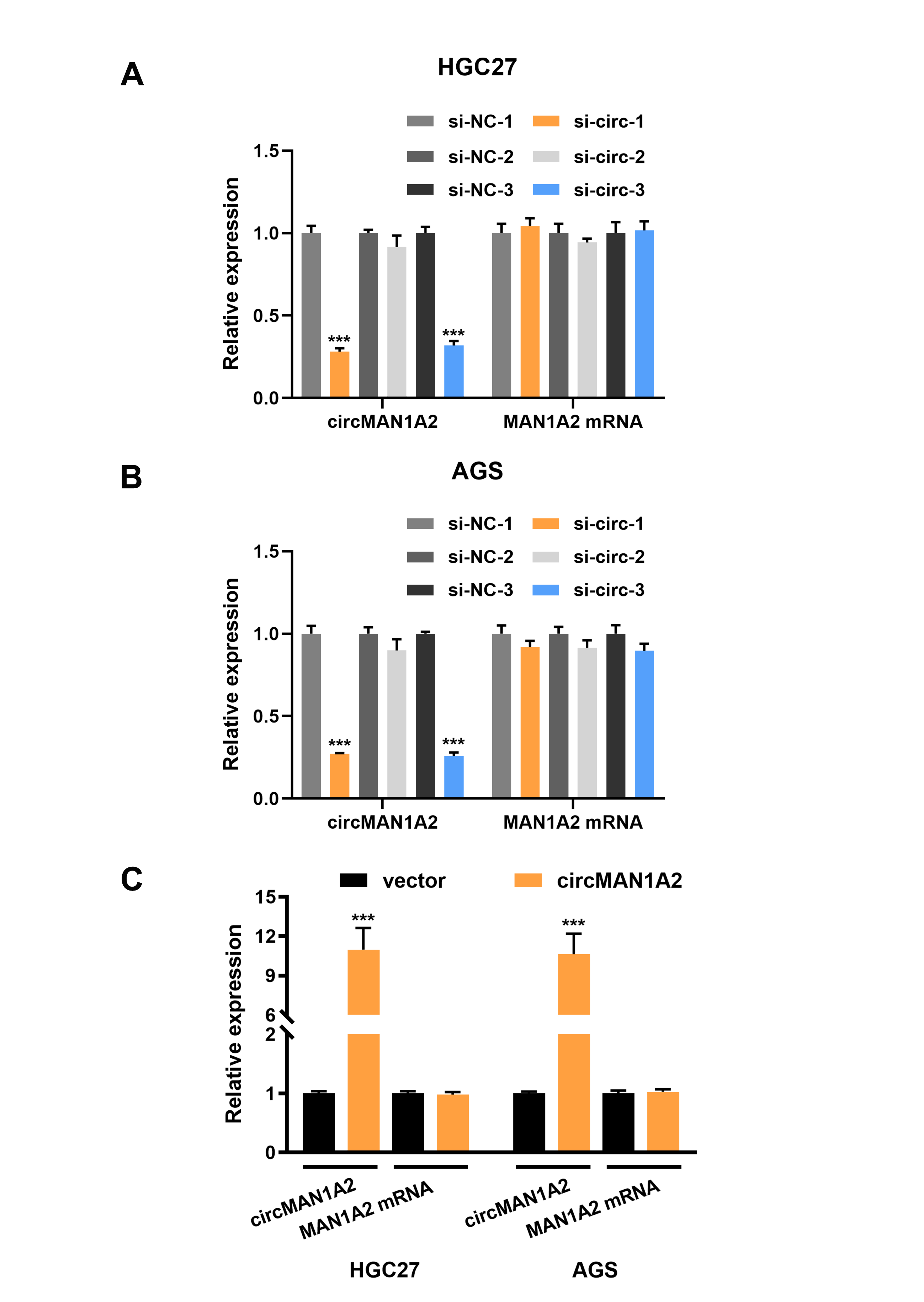

Supplement: Supplementary file 5 — Supplementary Material 5: Sup Fig. 3 A. Efficiency verification of three small interfering RNAs (si-RNAs) by qRT-PCR in HGC27 cells. B. Efficiency verification of three small interfering RNAs (si-RNAs) by qRT-PCR in AGS cells. C. Efficiency verification of circMAN1A2 overexpression plasmid by qRT-PCR in HGC27 and AGS cells. Graph represents mean ± SD; *p < 0.05, **p < 0.01, and ***p < 0.001. [file 13046_2025_3288_MOESM5_ESM.tif]

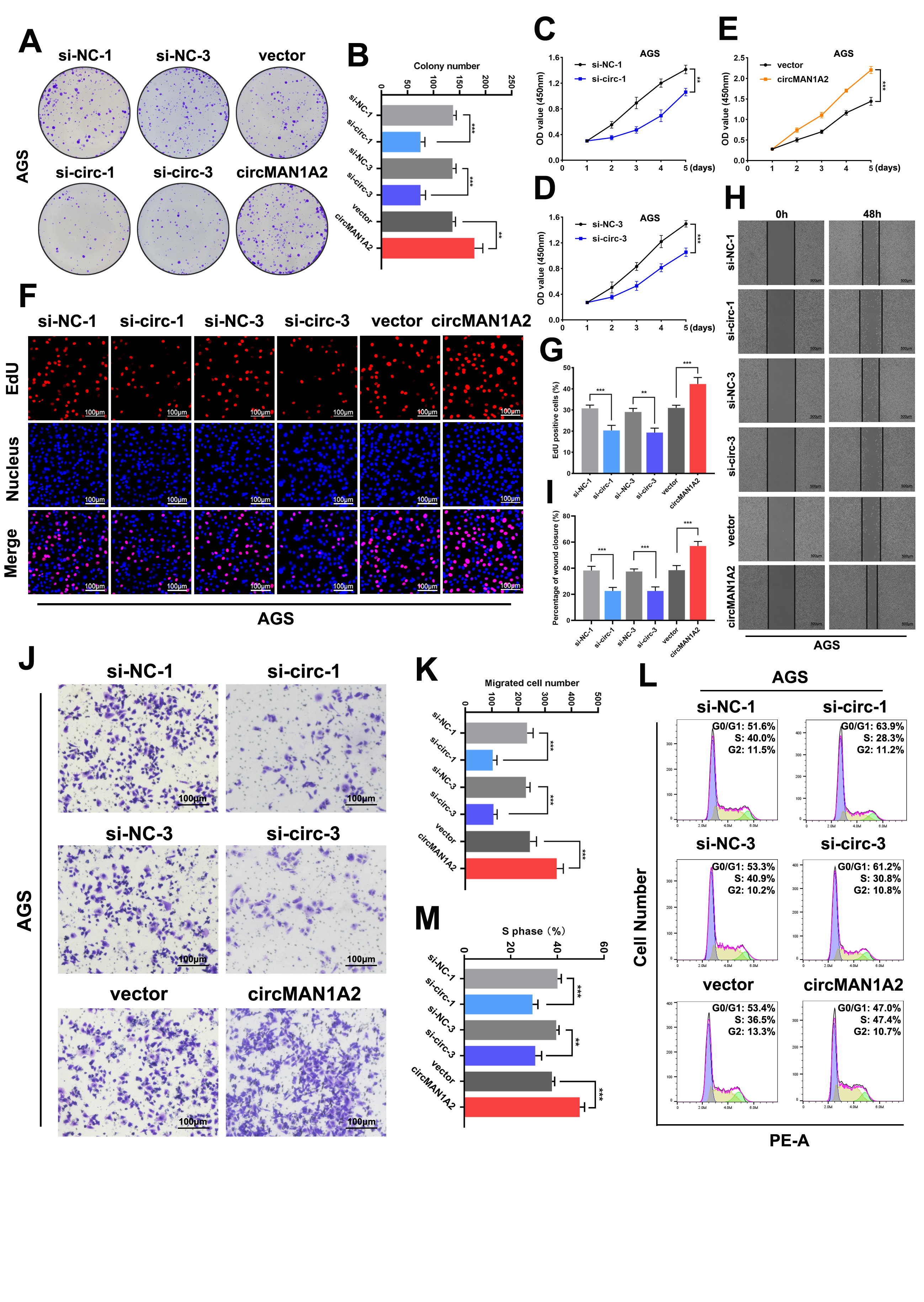

Supplement: Supplementary file 6 — Supplementary Material 6: Sup Fig. 4 A&B. The colony formation assay was performed to evaluate proliferation ability after upregulating or downregulating circMAN1A2 in AGS cells. C-E. The CCK8 assay was performed to evaluate proliferation ability after upregulating or downregulating circMAN1A2 in AGS cells. F&G. The EdU incorporation assay was performed to evaluate proliferation ability after upregulating or downregulating circMAN1A2 in AGS cells. Scale bar: 100 μm. H&I. The wound healing experiment was performed to evaluate migration ability after upregulating or downregulating circMAN1A2 in AGS cells. Scale bar: 500 μm. J&K. Transwell assay was performed to evaluate migration ability after upregulating or downregulating circMAN1A2 in AGS cells. Scale bar: 100 μm. L&M. The effect of circMAN1A2 on modulating AGS cell cycle progression was evaluated by flow cytometry assay. Graph represents mean ± SD; *p < 0.05, **p < 0.01, and ***p < 0.001. [file 13046_2025_3288_MOESM6_ESM.tif]

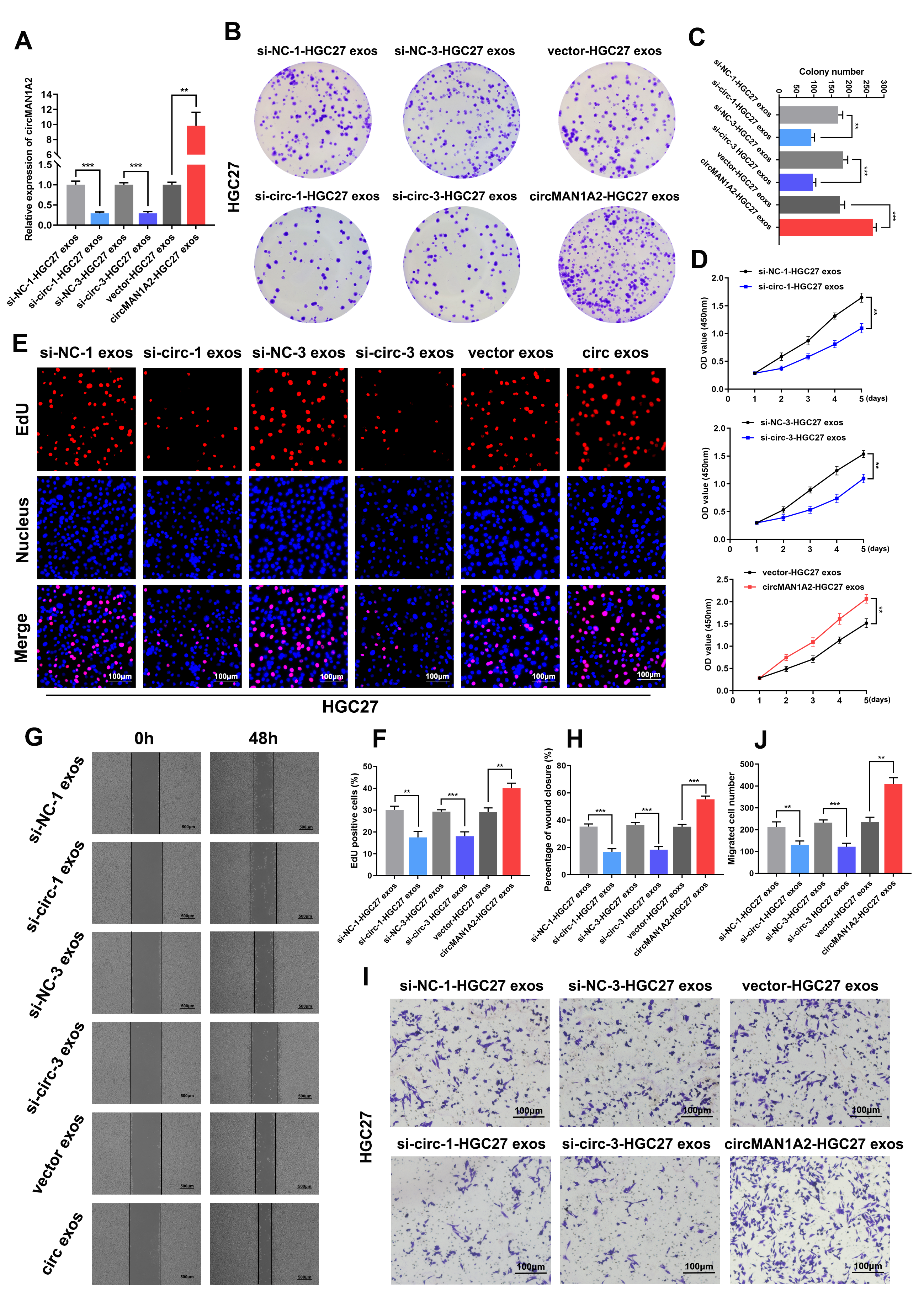

Supplement: Supplementary file 7 — Supplementary Material 7: Sup Fig. 5 A. Relative expression of circMAN1A2 in HGC27-derived exosomes from different treatment groups. B&C. The colony formation assay was performed to evaluate HGC27 cell proliferation ability after cocultured with exosomes from HGC27 cells upregulating or downregulating circMAN1A2. D. The CCK8 assay was performed to evaluate HGC27 cell proliferation ability after cocultured with exosomes from HGC27 cells upregulating or downregulating circMAN1A2. E&F. The EdU incorporation assay was performed to evaluate HGC27 cell proliferation ability after cocultured with exosomes from HGC27 cells upregulating or downregulating circMAN1A2. Scale bar: 100 μm. G&H. The wound healing experiment was performed to evaluate HGC27 cell migration ability after cocultured with exosomes from HGC27 cells upregulating or downregulating circMAN1A2. Scale bar: 500 μm. I&J. Transwell assay was performed to evaluate HGC27 cell migration ability after cocultured with exosomes from HGC27 cells upregulating or downregulating circMAN1A2. Scale bar: 100 μm. Graph represents mean ± SD; *p < 0.05, **p < 0.01, and ***p < 0.001. [file 13046_2025_3288_MOESM7_ESM.tif]

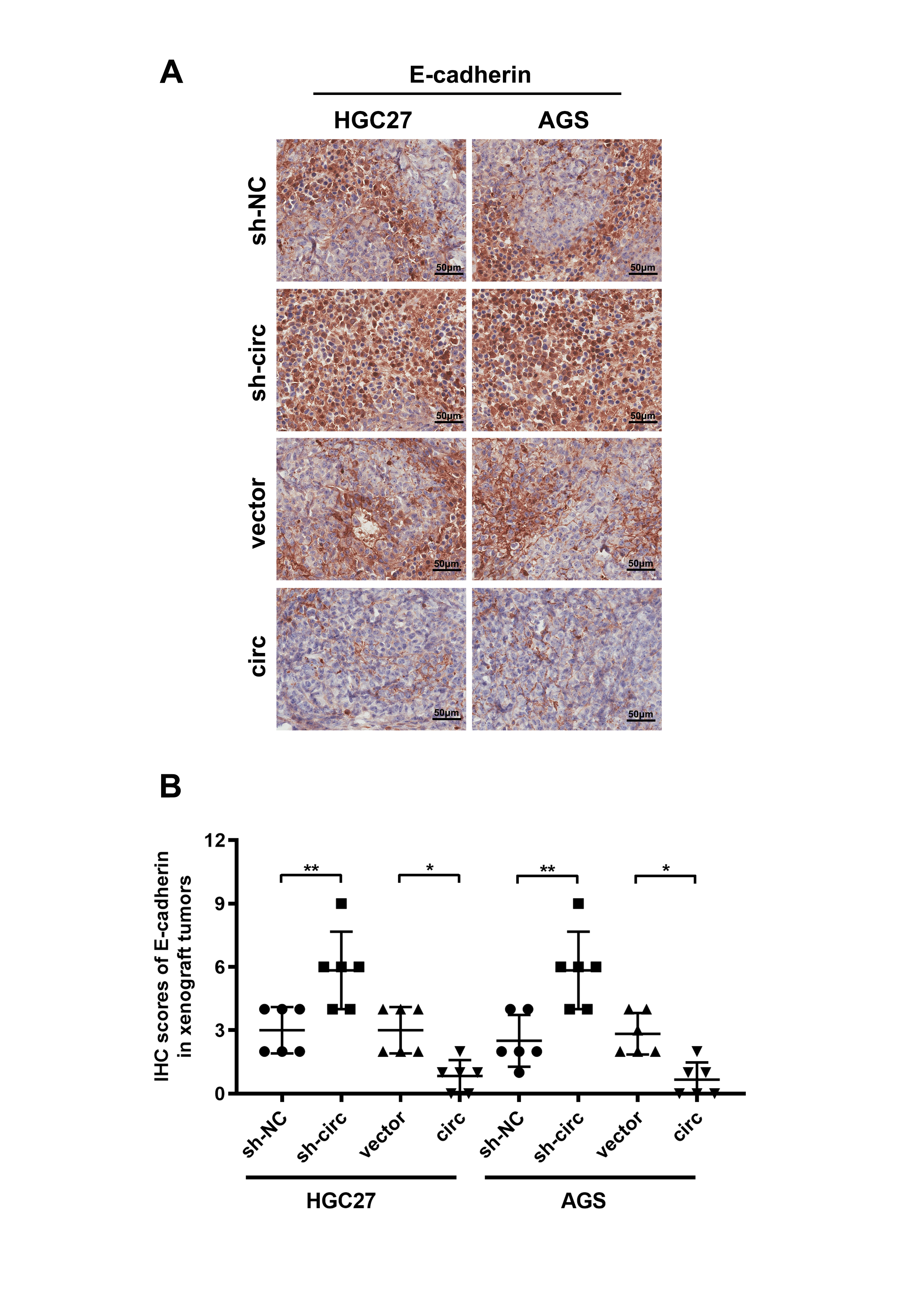

Supplement: Supplementary file 8 — Supplementary Material 8: Sup Fig. 6 A. Immunohistochemistry staining of E-cadherin in respective xenograft tumour tissues. Scale bar: 50 μm. B. IHC scores of E-cadherin in respective xenograft tumour tissues. Graph represents mean ± SD; *p < 0.05, **p < 0.01, and ***p < 0.001. [file 13046_2025_3288_MOESM8_ESM.tif]

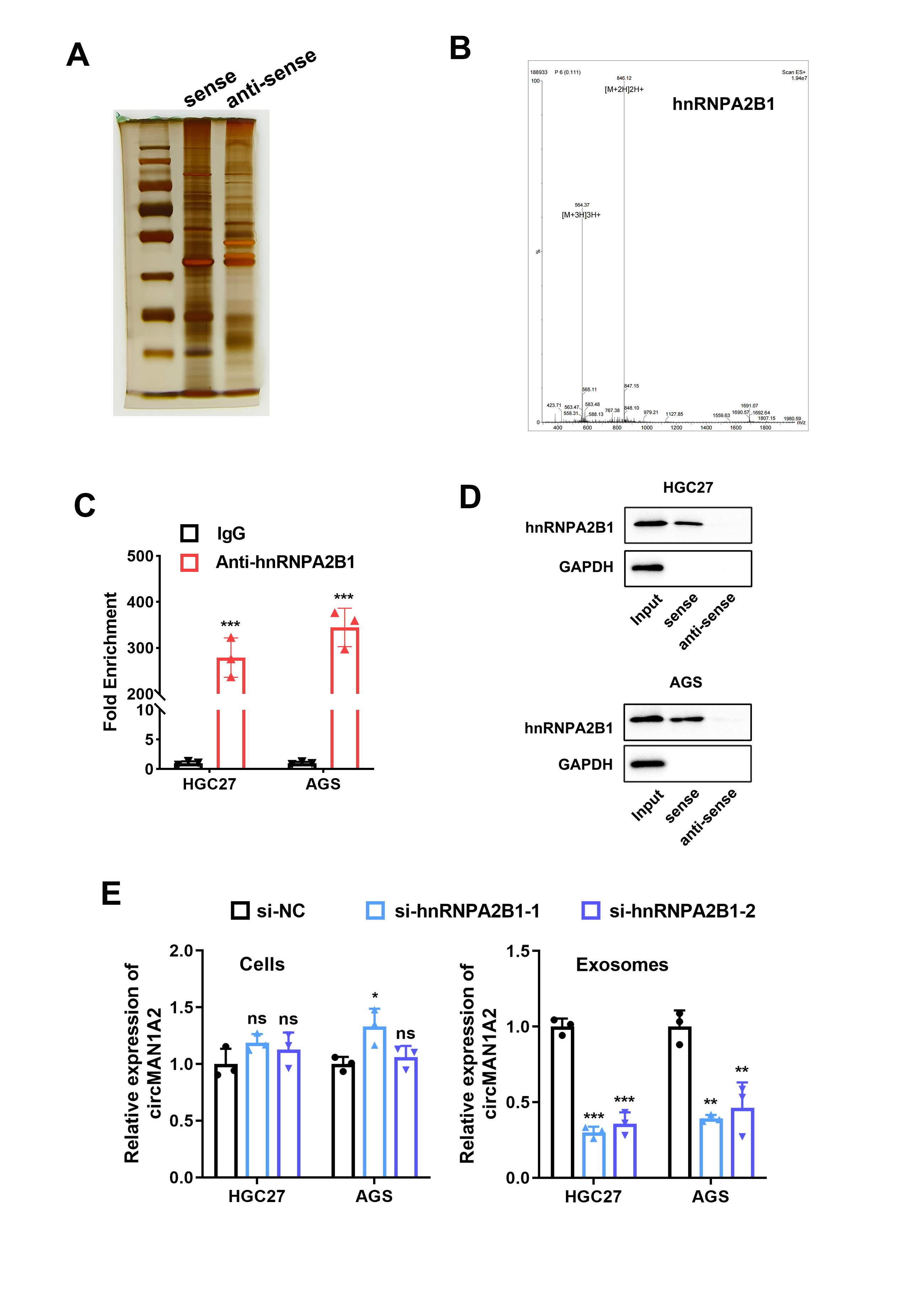

Supplement: Supplementary file 9 — Supplementary Material 9: Sup Fig. 7 A. Protein bands detected by silver stain for mass spectrometry of the circMAN1A2-protein complex pulled down by sense or anti-sense circMAN1A2 in HGC27 cells. B. The typical hnRNPA2B1 peptide was identified in circMAN1A2-enriched proteins based on MS analysis. C. RIP and qRT-PCR assays showed the interaction between hnRNPA2B1 and circMAN1A2 in HGC27 and AGS cells, using IgG and hnRNPA2B1 antibodies, n=3. D. RNA pull-down and Western blot assays were performed to confirm the interaction between hnRNPA2B1 and circMAN1A2 in HGC27 and AGS cells. E. qRT-PCR assays showed the relative expression levels of circMAN1A2 in HGC27 and AGS cells transfected with si-NC or si-hnRNPA2B1, and in exosomes extracted from HGC27 and AGS cells transfected with si-NC or si-hnRNPA2B1, n=3. Graph represents mean ± SD; *p < 0.05, **p < 0.01, and ***p < 0.001. [file 13046_2025_3288_MOESM9_ESM.tif]

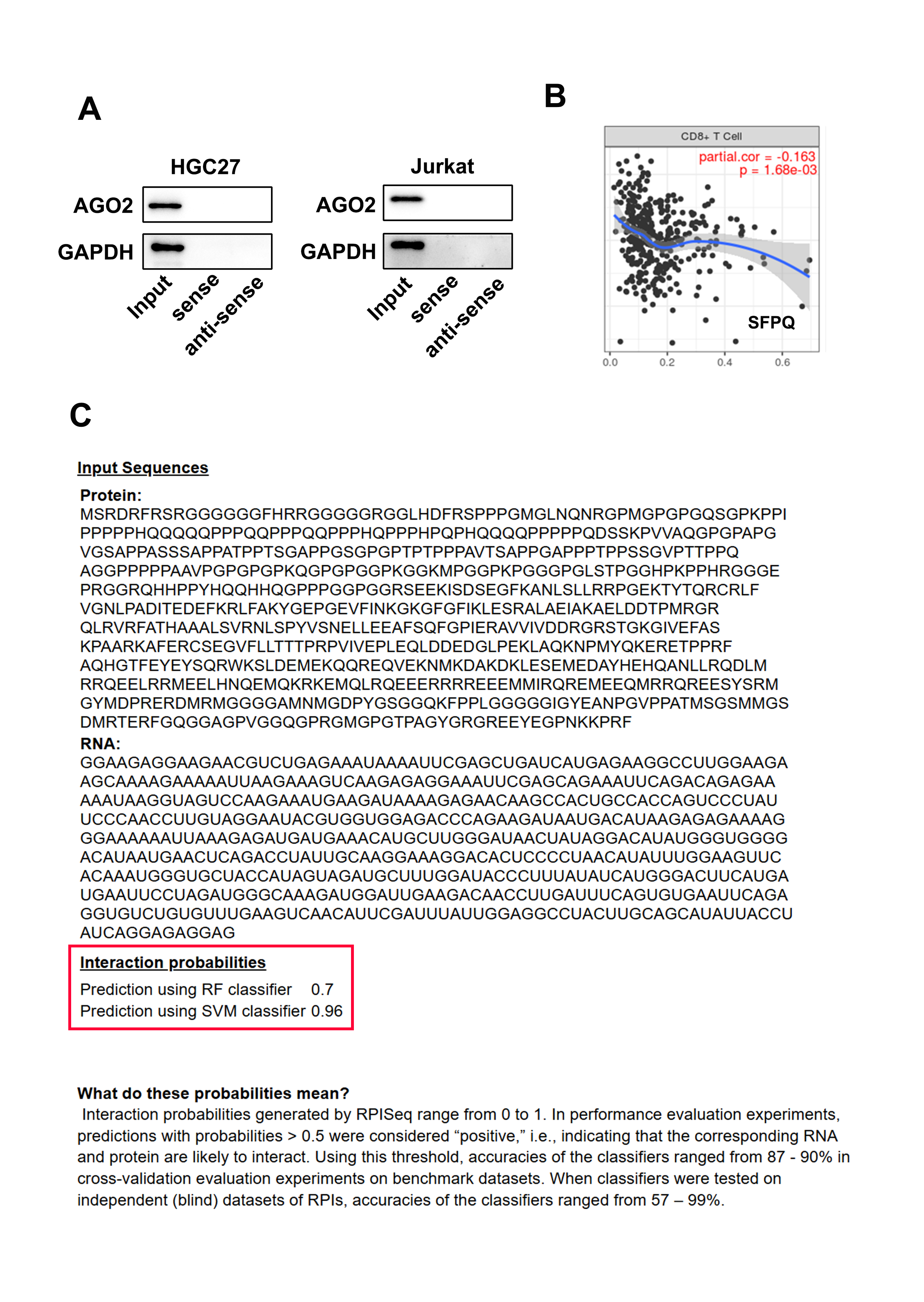

Supplement: Supplementary file 10 — Supplementary Material 10: Sup Fig. 8 A. RNA pull-down experiments were performed in HGC27 and Jurkat cells to detect AGO2 protein. B. Correlation of SFPQ with CD8+ T cell infiltration in GC. C. RPIseq prediction revealed the binding potential of SFPQ with circMAN1A2. [file 13046_2025_3288_MOESM10_ESM.tif]

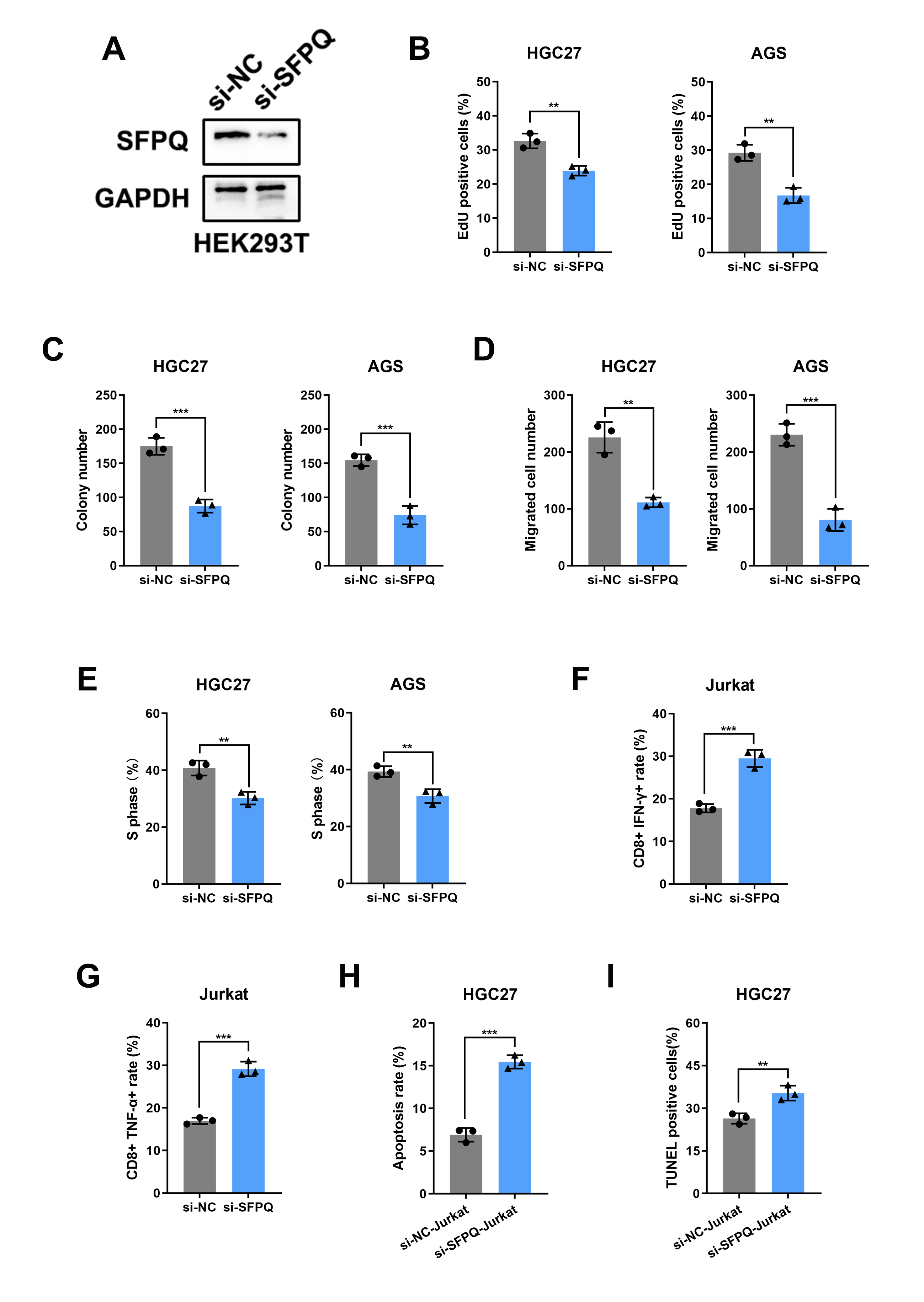

Supplement: Supplementary file 11 — Supplementary Material 11: Sup Fig. 9 A. Efficiency verification of si-SFPQ in HEK293T cells. B. Statistical graphs of EdU positive cells in HGC27 and AGS cells. C. Statistical graphs of the colony number in HGC27 and AGS cells. D. Statistical graphs of the migrated cell number in HGC27 and AGS cells. E. Statistical graphs of the S period proportion in the cell cycle process in HGC27 and AGS cells. F. Statistical graphs of CD8+ IFN-γ+ Jurkat cells. G. Statistical graphs of CD8+ TNF-α+ Jurkat cells. H. Statistical graphs of HGC27 apoptosis rates. I. Statistical graphs of TUNEL positive cells in HGC27. Graph represents mean ± SD; *p < 0.05, **p < 0.01, and ***p < 0.001. [file 13046_2025_3288_MOESM11_ESM.tif]

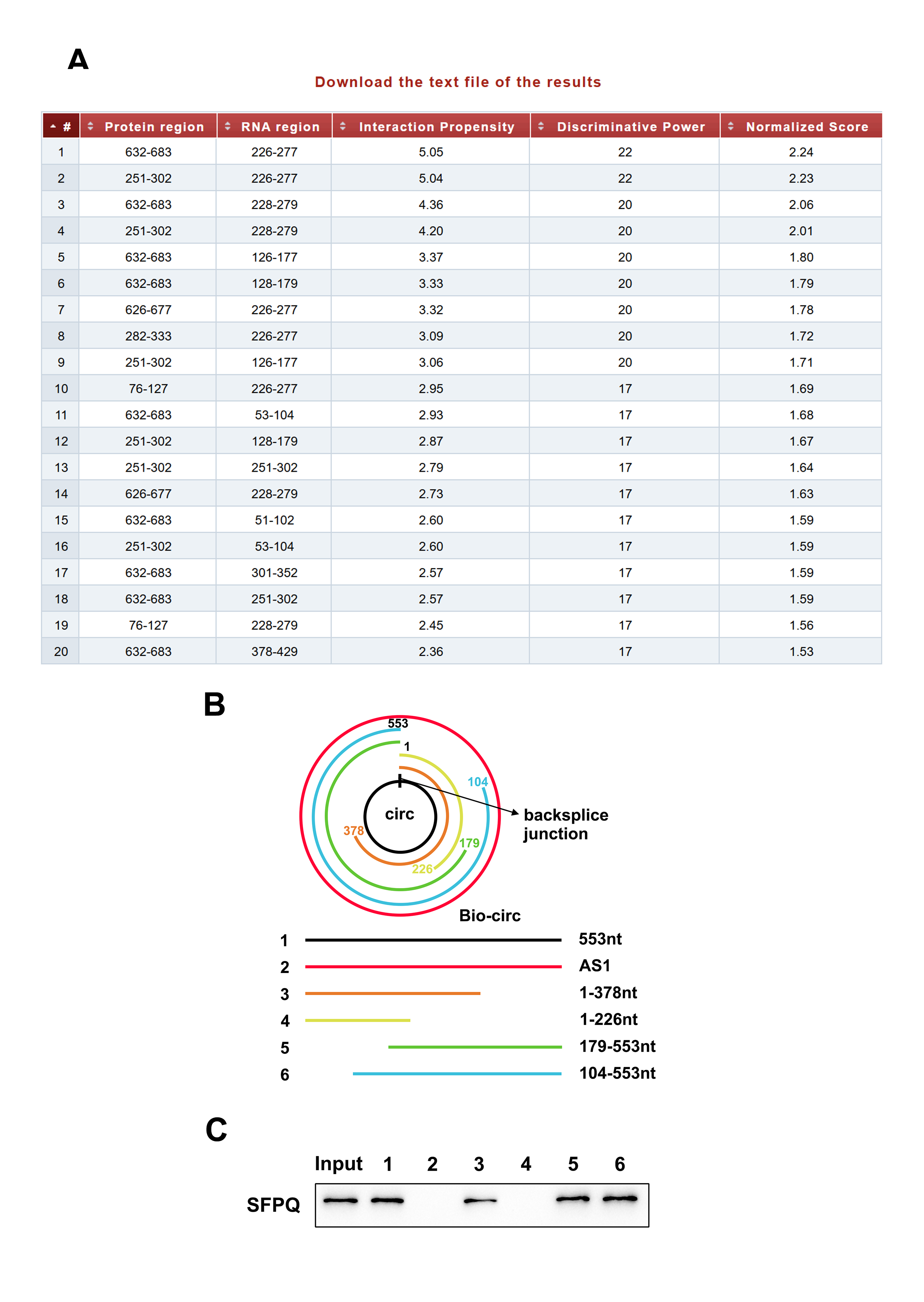

Supplement: Supplementary file 12 — Supplementary Material 12: Sup Fig. 10 A. The predicted regions in circMAN1A2 which exhibit the potential to bind to SFPQ. B. Different circMAN1A2 truncated probes were designed based on the potential binding regions. C. RNA pull-down and Western blot assays were performed to identify the regions binding to SFPQ. [file 13046_2025_3288_MOESM12_ESM.tif]

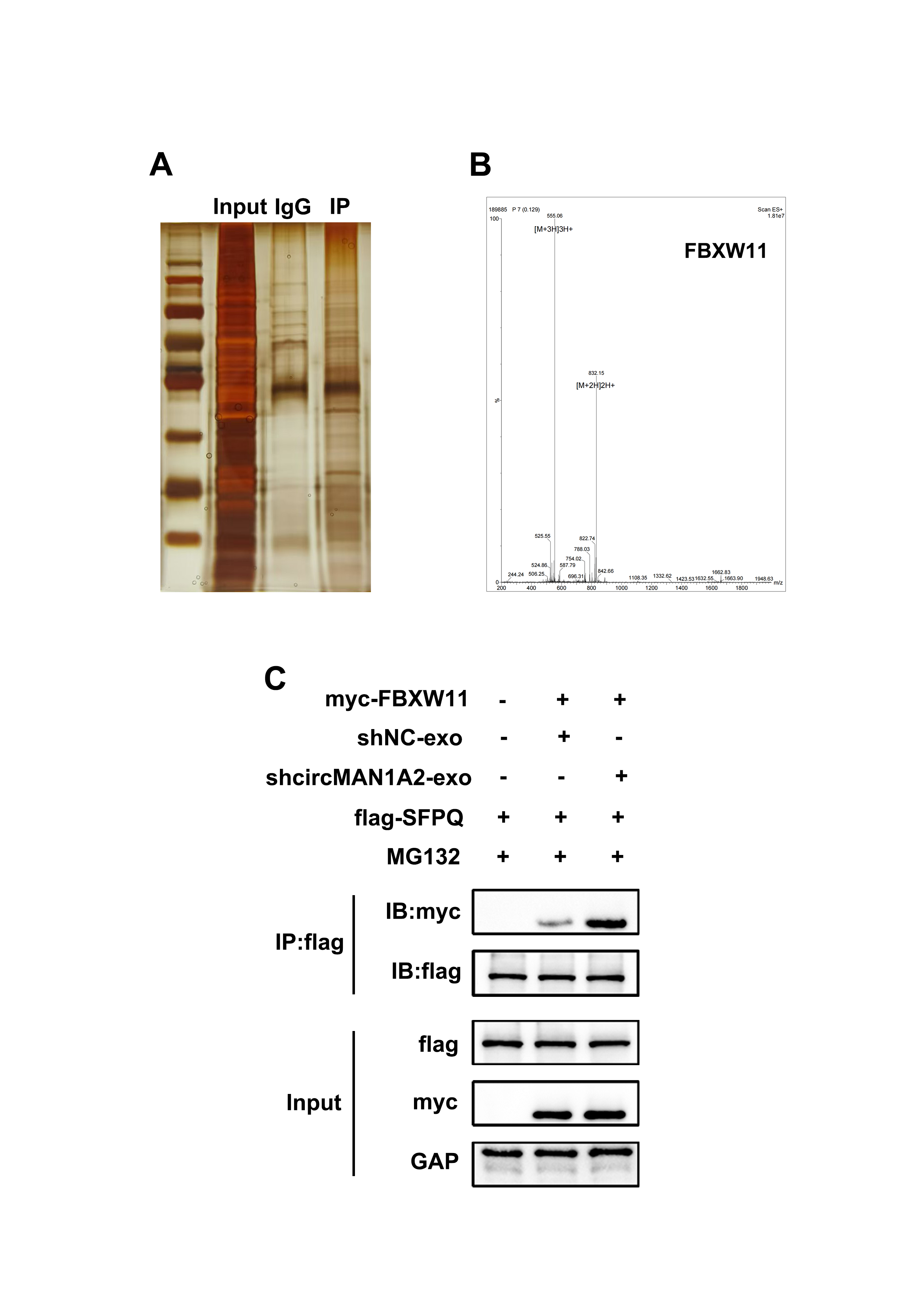

Supplement: Supplementary file 13 — Supplementary Material 13: Sup Fig. 11 A. Co-IP experiment was performed in HEK293T cells using SFPQ antibody. B. The typical FBXW11 peptide was identified in co-IP protein samples based on MS analysis. C. Co-IP assays confirmed that silencing circMAN1A2 increases the binding between FBXW11 and SFPQ. [file 13046_2025_3288_MOESM13_ESM.tif]
